# Supplementary material for: Mapping QTLs for enhancing early biomass derived from Aegilops tauschii in synthetic hexaploid wheat
Source: PLoS One. 2020 Jun 25;15(6):e0234882. doi: 10.1371/journal.pone.0234882 (PMC7316292; doi:10.1371/journal.pone.0234882)

PLOS ONE

Mapping QTL for enhancing early biomass derived from Aegilops tauschii in synthetic hexaploid wheat

--Manuscript Draft--

**Manuscript Number:**	PONE-D-20-04315
**Article Type:**	Research Article
**Full Title:**	Mapping QTL for enhancing early biomass derived from Aegilops tauschii in synthetic hexaploid wheat
**Short Title:**	Mapping QTL in synthetic hexaploid wheat
**Corresponding Author:**	Wuyun Yang<br>Crop Research Institute<br>Chengdu, CHINA
**Keywords:**	
**Abstract:**	Stronger early vigor plays a crucial role in wheat improvement by enhancing resource utilization efficiency. Synthetic hexaploid wheat (SHW) combining the elite genes of tetraploid wheat and Aegilops tauschii was widely applicated in wheat genetic improvement for its abundant genetic variation. The two SHWs Syn79 and Syn80 derived from the crosses of the same tetraploid wheat DOY1 with two different Ae. tauschii AT333 and AT428, respectively. And AT428 possessed better early vigor traits than AT333. To dissect their genetic basis on D genome, 203 recombinant inbred lines (RILs) derived from the cross Syn79 x Syn80 were developed and used to detect quantitative trait loci (QTL) for four early biomass related traits, including plant height (PH), tiller number (TN), shoot fresh weight (SFW) and shoot dry weight (SDW) per plant under five different environments. With the help of SNP markers, two genome regions on 1DS and 7D were detected stably to be association associated with early biomass, and exhibited pleiotropic effects on PH, TN, SFW and SDW. Four stable QTL for QPh.saas-1DS, QTn.saas-1DS, QSfw.saas-1DS and QSdw.saas-1DS were clustered in the region of 1DS from AX-94812958 to AX-110910133, and explained 7.92, 15.34, 9.64 and 10.15% of phenotypic variation on average, respectively. QPh.saas-7D, QTn.saas-7D, QSfw.saas-7D and QSdw.saas-7D were mapped in the interval of AX-109917900 - AX-110605376 on 7D, explaining the 16.12, 24.35, 15.25 and 13.37% of phenotypic variation on average, respectively. Moreover, the genomic regions on 1DS and 7D enhanced the early biomass by QTL alleles of the parent Syn80, which was from Ae. tauschii AT428. These findings suggest that the QTL enhancing early biomass from Ae. tauschii can express stably in hexaploid background at jointing stage and be used for wheat improvement.
**Order of Authors:**	Yumin Yang
	Hongshen Wan
	Fan Yang
	Chun Xiao
	Jun Li
	Meijin Ye
	Chunxiu Chen
	Guangmin Deng
	Qin Wang
	Aili Li
	Long Mao
	Wuyun Yang
	Yonghong Zhou

Powered by Editorial Manager® and ProduXion Manager® from Aries Systems Corporation

Additional Information:	
**Question**	**Response**
**Financial Disclosure**<br><br>Enter a financial disclosure statement that describes the sources of funding for the work included in this submission. Review the submission guidelines for detailed requirements. View published research articles from *PLOS ONE* for specific examples.<br><br>This statement is required for submission and **will appear in the published article** if the submission is accepted. Please make sure it is accurate.<br><br>**Unfunded studies**<br>Enter: *The author(s) received no specific funding for this work.*<br><br>**Funded studies**<br>Enter a statement with the following details:<br>• Initials of the authors who received each award<br>• Grant numbers awarded to each author<br>• The full name of each funder<br>• URL of each funder website<br>• Did the sponsors or funders play any role in the study design, data collection and analysis, decision to publish, or preparation of the manuscript?<br>• **NO** - Include this sentence at the end of your statement: *The funders had no role in study design, data collection and analysis, decision to publish, or preparation of the manuscript.*<br>• **YES** - Specify the role(s) played.<br><br>* typeset	This work was financially supported by National Natural Science Foundation of China (Grant No. 31661143007, 31401383) and Department of Science and Technology of Sichuan Province (Grant No. 2017JY0077, 2017JY0286).
**Competing Interests**<br><br>Use the instructions below to enter a competing interest statement for this submission. On behalf of all authors, disclose any competing interests that could be perceived to bias this work—acknowledging all financial support and any other relevant financial or non-financial competing interests.	The authors have declared that no competing interests exist.

This statement **will appear in the published article** if the submission is accepted. Please make sure it is accurate. View published research articles from *PLOS ONE* for specific examples.<br><br>**NO authors have competing interests**<br><br>Enter: *The authors have declared that no competing interests exist.*<br><br>**Authors with competing interests**<br><br>Enter competing interest details beginning with this statement:<br><br>*I have read the journal's policy and the authors of this manuscript have the following competing interests: [insert competing interests here]*<br><br>* typeset	
**Ethics Statement**<br><br>Enter an ethics statement for this submission. This statement is required if the study involved:<br><br>• Human participants<br>• Human specimens or tissue<br>• Vertebrate animals or cephalopods<br>• Vertebrate embryos or tissues<br>• Field research<br><br>Write "N/A" if the submission does not require an ethics statement.<br><br>General guidance is provided below. Consult the submission guidelines for detailed instructions. **Make sure that all information entered here is included in the Methods section of the manuscript.**	N/A

Powered by Editorial Manager® and ProduXion Manager® from Aries Systems Corporation

**Format for specific study types**<br><br>**Human Subject Research (involving human participants and/or tissue)**<br>• Give the name of the institutional review board or ethics committee that approved the study<br>• Include the approval number and/or a statement indicating approval of this research<br>• Indicate the form of consent obtained (written/oral) or the reason that consent was not obtained (e.g. the data were analyzed anonymously)<br><br>**Animal Research (involving vertebrate animals, embryos or tissues)**<br>• Provide the name of the Institutional Animal Care and Use Committee (IACUC) or other relevant ethics board that reviewed the study protocol, and indicate whether they approved this research or granted a formal waiver of ethical approval<br>• Include an approval number if one was obtained<br>• If the study involved *non-human primates*, add *additional details* about animal welfare and steps taken to ameliorate suffering<br>• If anesthesia, euthanasia, or any kind of animal sacrifice is part of the study, include briefly which substances and/or methods were applied<br><br>**Field Research**<br><br>Include the following details if this study involves the collection of plant, animal, or other materials from a natural setting:<br>• Field permit number<br>• Name of the institution or relevant body that granted permission	
**Data Availability**<br><br>Authors are required to make all data underlying the findings described fully available, without restriction, and from the time of publication. PLOS allows rare exceptions to address legal and ethical concerns. See the PLOS Data Policy and FAQ for detailed information.	Yes - all data are fully available without restriction

A Data Availability Statement describing where the data can be found is required at submission. Your answers to this question constitute the Data Availability Statement and **will be published in the article**, if accepted.<br><br>**Important:** Stating 'data available on request from the author' is not sufficient. If your data are only available upon request, select 'No' for the first question and explain your exceptional situation in the text box.<br><br>Do the authors confirm that all data underlying the findings described in their manuscript are fully available without restriction?	
**Describe where the data may be found in full sentences. If you are copying our sample text, replace any instances of XXX with the appropriate details.**<br><br>• If the data are **held or will be held in a public repository**, include URLs, accession numbers or DOIs. If this information will only be available after acceptance, indicate this by ticking the box below. For example: *All XXX files are available from the XXX database (accession number(s) XXX, XXX.).*<br>• If the data are all contained **within the manuscript and/or Supporting Information files**, enter the following: *All relevant data are within the manuscript and its Supporting Information files.*<br>• If neither of these applies but you are able to provide **details of access elsewhere**, with or without limitations, please do so. For example:<br><br>*Data cannot be shared publicly because of [XXX]. Data are available from the XXX Institutional Data Access / Ethics Committee (contact via XXX) for researchers who meet the criteria for access to confidential data.*<br><br>*The data underlying the results presented in the study are available from (include the name of the third party*	All relevant data are within the manuscript and its Supporting Information files.

and contact information or URL).<br>• This text is appropriate if the data are owned by a third party and authors do not have permission to share the data.<br><br>* typeset	
Additional data availability information:	

Manuscript Click here to download Manuscript Manuscript.doc

Mapping QTL for enhancing early biomass derived from *Aegilops tauschii* in synthetic hexaploid wheat

Yumin Yang[1,2,4,¶], Hongshen Wan[3,4,¶], Fan Yang[1], Chun Xiao[2], Jun Li[3,4], Meijin Ye[1], Chunxiu Chen[2], Guangmin Deng[2], Qin Wang[3,4], Aili Li[5], Long Mao[5], Wuyun Yang[3,4*], Yonghong Zhou[1*]

[1] Triticeae Research Institute, Sichuan Agricultural University, Chengdu, China
[2] Soil and Fertilizer Research Institute, Sichuan Academy of Agricultural Sciences, Chengdu, China
[3] Crop Research Institute, Sichuan Academy of Agricultural Sciences, Chengdu, China
[4] Key Laboratory of Wheat Biology and Genetic Improvement on Southwestern China (Ministry of Agriculture and Rural Areas), Chengdu, China
[5] Institute of Crop Sciences, Chinese Academy of Agricultural Sciences, Beijing, China

***** Corresponding author

E-mail addresses: yangwuyun@126.com (W. Yang), zhouyhdavid@126.com (Y. Zhou)

[¶] These authors contributed equally to this work.

Abstract

Stronger early vigor plays a crucial role in wheat improvement by enhancing resource utilization efficiency. Synthetic hexaploid wheat (SHW) combining the elite genes of tetraploid wheat and *Aegilops tauschii* was widely applicated in wheat genetic improvement for its abundant genetic variation. The two SHWs Syn79 and Syn80 derived from the crosses the same tetraploid wheat DOY1 with two different *Ae. tauschii* AT333 and AT428, respectively. And AT428 possessed better early vigor traits than AT333. To dissect their genetic basis on D genome, 203 recombinant inbred lines (RILs) derived from the cross Syn79 x Syn80 were developed and used to detect quantitative trait loci (QTL) for four early biomass related traits, including plant height (PH), tiller number (TN), shoot fresh weight (SFW) and shoot dry weight (SDW) per plant under five different environments. With the help of SNP markers, two genome regions on 1DS and 7D were detected stably to be association with early biomass, and exhibited pleiotropic effects on PH, TN, SFW and SDW. Four stable QTL for *QPh.saas-1DS*, *QTn.saas-1DS*, *QSfw.saas-1DS* and *QSdw.saas-1DS* were clustered in the region of 1DS from *AX-94812958* to *AX-110910133*, and explained 7.92, 15.34, 9.64 and 10.15 % of phenotypic variation on average, respectively. *QPh.saas-7D*, *QTn.saas-7D*, *QSfw.saas-7D* and *QSdw.saas-7D* were mapped in the interval of *AX-109917900- AX-110605376* on 7D, explaining the 16.12, 24.35, 15.25 and 13.37 % of phenotypic variation on average, respectively. Moreover, the genomic regions on 1DS and 7D enhanced the early biomass by QTL alleles of the parent Syn80, which was from *Ae. tauschii* AT428. These findings suggest that the QTL enhancing early biomass from *Ae.*

tauschii can express stably in hexaploid background at jointing stage and be used for wheat improvement.

Introduction

Common wheat (*Triticum aestivum* L., 2n = 6x = 42, AABBDD), as an important food crop in the world, originated from the spontaneous hybridization of tetraploid *Triticum turgidum* wheat (2n = 4x = 28, AABB) with diploid *Aegilops tauschii* Coss (2n = 2x = 14, DD) [1,2]. It is believed that only a few accessions of the donor species were involved in the evolution of common wheat, especially for the D genome donor. Consequently, the genetic diversity of common wheat was significantly decreased in comparison with its donor species. Due to the evolutionary bottleneck, most of the genetic variation in *Ae. tauschii* did not exist in the commonly available hexaploid germplasm [3], and only 7 % of the variants observed in *Ae. tauschii* were reserved in common wheat [4,5]. To enhance the transferring efficiency of elite genes from *Ae. tauschii* species to common wheat, scientists created synthetic hexaploid wheat (SHW) from crosses between *T. turgidum* and *Ae. tauschii* to broaden the genetic variation of hexaploid wheat [6]. Over 1000 SHW lines have been produced by using more than 600 *Ae. tauschii* accessions stored at the International Maize and Wheat Improvement Center (CIMMYT; Mexico City, Mexico) [7]. SHWs with huge genetic diversity have shown their outstanding superiority in resistance to diseases and pests, tolerance to environmental stresses, and desirable quantitative traits, and been used widely in

common wheat breeding [8-14]. Chinese scientists have shown a high interest in CIMMYT SHW since the early 1990s [15-18]. More than 200 CIMMYT SHW accessions have been introduced into China in 1995 [14] . In recent years, several commercial wheat varieties have been also created and released in China [9,14,19]. Furthermore, several favorable introgressions from *Ae. tauschii* have been identified in synthetic derivatives [19]. A major QTL on 4DL associated with leaf sheath hairiness in a synthetic derivative wheat variety Chuanmai42 was detected, and its wild allele ~~was~~ originated from *Ae. tauschii*, which ~~was~~ significantly increased grain weight, grain yield, and yield related characters [20].

Stronger vigor cultivars have advantages to enhance the population water-use efficiency by shading the soil surface faster and reducing evaporative losses from the soil [21-23]. Rapid early development of leaf area and root system are associated with increased water and nutrient use efficiency, rates of light interception and biomass resulting drought tolerance and high yield potential [22, 23]. In recent years, we have screened the biomass of CIMMYT SHWs and found two SHWs (Syn79 and Syn80) derived from the same teraploid wheat (durum wheat DOY1) and two different *Ae. tauschii* accessions having significant different biomass in the whole developing stage, and the significant difference of biomass between them was caused by their different D genome donors. And the growth, nutrient accumulation, nutrient distribution and utilization of Syn79 and Syn80 were significantly different under different environments [24]. In order to evaluate the genetic impact of the different D genomes on early vigor in hexaploid wheat, a population of recombinant inbred lines (RILs)

derived from a cross between Syn79 and Syn80 have been developed. The goal of this study was to map the major QTL associated with early biomass from *Ae. tauschii* under hexaploid wheat background at jointing stage, for the molecular improvement of wheat yield with SHW.

Materials and methods

Plant materials

Two hundred and three F_9 recombinant inbred lines (RILs) derived from Syn79 x Syn80 cross and their parents were used for QTL mapping in this study. Syn79 and Syn80 were generated from durum wheat DOY1 (2n=28, AABB) crossing with *Ae. tauschii* (2n=14, DD) by CIMMYT [6]. The AB genome of Syn79 and Syn80 was from the same durum donor DOY1, while their D genomes were from two different *Ae. tauschii* accessions(AT333 and AT428). Syn80 had stronger early vigor than Syn79 (Fig 1), which was caused by their different D genome, and AT428 possessed better early vigor traits than AT333.

Fig 1. Early growth of the two parents and their RILs in the jointing stage.

Field trials

A total of five trials for Syn79, Syn80 and 203 RILs were conducted at Guang-Han Station (GHS) in 2017-2019 (2017GHS, 2018GHS, 2019GHS) and Cang-Shan Station (CSS) in 2017 and 2018 (2017CSS, 2018CSS). GHS and CSS of Sichuan Academy of Agricultural

Sciences (SAAS) are the representative of the plain region and hilly region in Sichuan province, respectively. Chemical properties of their soils of five trials were shown in Table 1. The organic matter, total nitrogen and available nitrogen of soil in GHS were significantly higher than those in CCS, and the total potassium of soil in CSS was more than that in GHS (Table 1).

Table 1. Chemical properties of soil in different field trials.

Trials	pH	Organic matter (g/kg)	Total nitrogen (g/kg)	Total phosphorus (g/kg)	Total potassium (g/kg)	Available nitrogen (mg/kg)	Available phosphorus (mg/kg)	Available potassium (mg/kg)
2017GHS	6.84	31.9	1.99	0.723	16.25	165	6.9	90
2018GHS	6.45	39.7	2.31	0.860	18.77	206	15.8	105
2019GHS	6.71	28.9	2.03	0.674	19.00	183	11.0	96
2017CSS	7.81	9.5	0.77	0.556	23.81	47	3.7	100
2018CSS	8.24	15.7	1.23	0.328	22.90	97	2.9	137

The trials were performed in randomized complete blocks with three replicates. Each plot had five 1.5 m rows spaced 0.5 m apart. At two-leaf stage, only ten evenly distributed plants in each row were retained for further growth. Field management consisted of commonly under-taken practices in wheat production.

Trait evaluation

Four early biomass related traits, plant height (PH), tiller number (TN), shoot fresh weight (SFW) and shoot dry weight (SDW) per plant were investigated RILs and their parents at the jointing stage. In each plot, 10 plants with whole tissues including root and shoot were randomly selected to evaluate traits associated with early biomass. Sampling at

the jointing stage, plants at the ends of each row were disregarded to minimize within-row edge effects. After sampling, the roots of the 10 plants were cut, PH, TN SFW and SDW were measured. All traits were described by the mean values of 10 plants for corresponding line. The measurement for SFW was accomplished within 12 hours after sampling.

SNP Genotyping

A total of 50 mg of plant fresh leaves was collected from 2-week-old seedlings and DNA was extracted using the NuClean Plant Genomic DNA Kit (CWBio, Beijing, China). Eluted DNA was quantified using Qubit 4 Fluorometer (Life Technologies Holdings Pte Ltd, Singapore) and then normalized using a 12-channel electronic pipette with a volume range of 10 to 100μL (Eppendorf, Hamburg, Germany) to obtain the concentration required for genotyping.

The RILs and their parents Syn79 and Syn80 were executed on the Affymetrix platform of Axiom Wheat Breeder's Genotyping Array with 13947 SNP markers including 1272 functional markers by China Golden Marker Biotech Co Ltd (Beijing, China). The collected fluorescence signal from SNP array ~~were~~ processed and analyzed by the functions of apt-genotype-axiom for genotype calling, ps-metrics for generating various QC metrics and ps-classification for classifying SNPs in the software of Affymetrix Axiom Analysis Suite version 4.0.1. Among 13947 SNP markers, a total of 3480 SNPs is distributed on D genome, and was used for parental polymorphic analysis.

Statistical and QTL analysis

Descriptive analysis, analysis of variance (ANOVA) and correlation analysis for the phenotypic data were calculated using the SPSS statistical package (SPSS Inc., Chicago, IL). Variation of genotypes for phenotypic traits were evaluated using mean, stansard deviation (SD), coefficient of variation (CV), maximum (Max) and minimum (Min).Variance components of ANOVA for all traits was calculated based the general linear model (GLM) to detected the effect of genotypes, environments and genotypes × environments interaction. Broad sense heritability (H^2) was estimated with the formula: $H^2= \sigma^2g/ (\sigma^2g + \sigma^2ge/n + \sigma^2e/nr)$, where σ^2g is genetic variance, σ^2ge is the variance of genotype-environment interaction, σ^2e is experiment error variance, n is the number of trials, r is the number of replications.

The QTL IciMapping Software version 4.1 [25,26] was used for genetic linkage map construction. The location of SNP marker was aligned according to the physical map of *Ae. tauschii* AL8/78 for D genome [27]. The genetic linkage map was constructed according to 153 polymorphic markers between Syn79 and Syn80 (the parents), which were screened from 3480 SNP markers distributed on D genome. The map covered over 803.84 cM on wheat D genome, with an average distance of 5.25 cM between adjacent polymorphic markers.

QTL analysis for the measured traits under the five different environmental conditions were performed using the inclusive composite interval mapping (ICIM) option on the QTL IciMapping Software version 4.1. Significant LOD threshold was determined by 1000

permutations and P = 0.05. Linked QTL with the genetic distance less than 20 cM were considered as one single QTL, which were named according to Ayalew et al. [28].

Results

Phenotypic analysis

Five different field trials were conducted at two locations over 3 years to evaluate early biomass related traits of the RIL population as well as their parents Syn79 and Syn80. Syn80 have greater early biomass than Syn79 (Fig 1). The values of PH, TN, SFW and SDW for Syn80 were significantly larger than those of Syn79 under all five environments (Table 2). Independent of the differences between the two parents, in all trials there were significant variations in the investigated traits of the RIL populations, with values spanning much larger ranges than those defined by the parental values. And the phenotypic data was distributed normally in the RILs (Fig 2). Variation of phenotypic data was tremendous in the RILs, especially for SFW and SDW. And variation was determined by genotypes, environments and genotypes × environments interaction. Their heritability ranged from 39.20 to 43.27 % (Table 2). It suggested that those phenotypic traits were controlled by multigene, and these genes' phenotypic traits were significantly affected by environment .

Fig 2. Distribution graph of the phenotypic data for PH, TN, SFW and SDW.

Table 2. Parental values, population distribution parameters, and heritability of the investigated traits

Trait	Environment	Parents		RILs			H^2 (%)	F-values from ANOVA		
		Syn79	Syn80	Mean±SD	CV(%)	Min-Max		Environment	Genotype	Environment×genotype
PH (cm)	2017GHS	31.87	48.64**	38.43±5.98	15.56	27.00-59.20	43.27	1368.74**	14.05**	2.02**
	2018GHS	34.48	53.81**	50.12±8.07	16.10	29.38-65.33				
	2019GHS	46.11	63.33**	60.87±8.20	13.47	38.11-81.94				
	2017CSS	42.00	56.33**	54.40±8.34	15.33	27.17-69.33				
	2018CSS	49.67	65.11**	59.84±9.07	15.16	27.58-76.56				
TN (No./plant)	2017GHS	5.60	12.53**	10.01±2.58	25.77	4.50-17.90	43.11	780.24**	14.32**	2.05**
	2018GHS	8.17	16.67**	15.05±3.92	26.05	6.00-23.00				
	2019GHS	10.00	15.78**	14.23±3.73	26.21	5.78-21.67				
	2017CSS	7.00	12.00**	9.22±2.58	27.98	2.00-15.00				
	2018CSS	8.33	13.22**	11.16±3.31	29.66	2.33-18.33				
SFW (g/plant)	2017GHS	17.09	73.72**	48.25±25.60	53.06	7.02-136.54	40.20	144.40**	14.05**	2.16**
	2018GHS	21.79	70.78**	60.92±29.91	49.10	7.30-156.94				
	2019GHS	29.40	78.37**	64.94±29.89	46.03	10.97-169.70				
	2017CSS	26.58	82.57**	52.69±25.50	48.40	3.59-132.55				
	2018CSS	36.89	96.08**	75.44±39.27	52.05	4.72-158.86				
SDW (g/plant)	2017GHS	2.85	10.22**	6.64±3.34	50.30	1.04-16.19	39.20	161.47**	84.08**	6.60**
	2018GHS	3.43	10.24**	8.35±3.58	42.87	1.04-20.54				
	2019GHS	4.63	11.87**	8.96±4.31	48.10	1.43-17.80				
	2017CSS	4.43	11.45**	7.25±3.28	45.24	0.46-18.13				
	2018CSS	5.59	13.98**	10.18±4.95	48.62	0.69-20.57				

* And ** indicate significant differences at P= 0.05 and 0.01, respectively.

Correlations among the PH, TN, SFW and SDW in different trials are presented in Table 3. It showed that significantly positive correlations among these traits were detected in early growth stage. Their average coefficients in five trials ranged from 0.581 to 0.975. The PH was significantly positively correlated with TN, and the coefficients ranged from 0.424 to 0.683 in different trials (Table 3). Both PH and TN showed significant positive correlations with SFW and SDW, and the coefficients were higher than that between PH and TN. These results suggested that greater early biomass was related with higher PH, more TN, heavier SFW and SDW, and the gene related to early biomass had pleiotropic effects on all four traits.

Table 3. Correlation coefficients between the four traits in RILs in different trials.

	TN	SFW	SDW
PH	O.673**	0.724**	0.733**
	0.424**	0.364**	0.355**
	0.683**	0.784**	0.808**
	0.606**	0.821**	0.835**
	0.612**	0.791**	0.791**
TN		0.708**	0.711**
		0.142*	0.141*
		0.731**	0.728**
		0.668**	0.678**
		0.749**	0.713**
SFW			0.967**
			0.969**
			0.978**
			0.983**
			0.980**

*And** indicate significance at P=0.05 and 0.01 level, respectively.

Genetic map of D genome

In this study, we used Wheat Breeder's Genotyping Array to genotype the A, B and D genome, respectively. For AB genome, a total of 10467 SNP labels anchored on the genotyping array were used to check the genotype of AB genome of RIL population and their parents, which were generated from the same AB donor. ~~And~~ the results showed that almost all SNP markers on AB genome had no polymorphism between two parents. For D genome, 3480 SNP labels were selected to fix on the chip by China Golden Marker Biotech Co Ltd (Beijing, China). Among these scanned markers, 153 markers on D genome had polymorphism between two parents, and distributed unequally on the seven chromosomes of the D genome (Table 4). The number of polymorphic markers on different chromosomes ranged from 8 on 3D to 34 on 7D (Table 4).

Table 4. SNP markers on the D genome.

Parameter	1D	2D	3D	4D	5D	6D	7D	Total
Total Makers	370	634	536	265	550	428	697	3480
Polymorphic markers	30	20	8	21	25	15	34	153
Polymorphism rate (%)	8.11	3.15	1.49	7.92	4.55	3.50	4.88	4.40
Map length (cM)	127.58	69.22	21.35	145.52	135.72	89.95	214.50	803.84
Distance between polymorphic markers (cM)	4.25	3.46	2.67	6.93	5.43	6.00	6.31	5.25

For linkage map construction, SNP markers were grouped according to their anchored chromosomes of *Ae. tauschii* AL8/78 for D genome, and then aligned by nnTwoOpt method. The whole genetic map covered over 803.84 cM on D genome with an average distance between adjacent markers was 5.25 cM (Table 4). There were no genetic gaps with adjacent marker distance more than 50 cM in each chromosome, and the average distance between two adjacent markers ranged from 2.67 cM to 6.93 cM. For all the 7 chromosomes, their linkage maps ranged from 21.35 cM to 214.50 cM. For the chromosome 2D and 3D, the total distance of the linkage maps constructed by this population and the Wheat Breeder's Genotyping Array were 69.22 cM and 21.35 cM, respectively. Out of the genomic regions of linkage maps, no polymorphic markers were detected by this SNP array.

Genotypic markers were tested for segregation distortion (deviation expected 1:1 ratios) by Chi-squared tests. Among the 153 SNP loci, 54 loci showed segregation distortion in RILs (Table 5). Almost all loci were biased to Syn80 with larger early biomass, which means that in those loci most of the RILs progeny preferentially inherited the female parent Syn80. And only four loci were male-biased (Table 5). Among those female-biased loci, the number of loci on different chromosomes distributed from 1 on 3D to 18 on 7D. And three genomic regions were detected to be Syn80-biased on chromosome 1D, 2D and 7D (Table 5), and covered about 50, 20 and 40 cM on 1D, 2D and 7D, respectively.

Table 5. Segregation distortion of SNP loci in RILs

Chromosome	Syn80-biased Locus		Unbiased Locus		Syn79-biased Locus	
	Number	Rate (%)	Number	Rate (%)	Number	Rate (%)
1D	11	36.67	19	63.33	0	0.00

2D	14	70.00	5	25.00	1	5.00
3D	1	12.50	7	87.50	0	0.00
4D	2	9.52	19	90.48	0	0.00
5D	3	12.00	20	80.00	2	8.00
6D	1	6.67	14	93.33	0	0.00
7D	18	52.94	15	44.12	1	2.94
Total	50	32.68	99	64.71	4	2.61

Underline means genetic regions with linked loci; Chi-squared test were programmed at P=0.05 level

QTL on D genome

With the linkage map constructed by 153 SNP markers on D genome, QTL for PH, TN, SFW and SDW were identified under five environmental conditions using the inclusive composite interval mapping (ICIM) program.

PH and TN are common agronomic traits in wheat, and the higher PH and TN in seeding growth stage are positively correlated with enhanced water-use efficiency of the population by shading the soil surface faster and reducing evaporative losses from the soil. A total of two QTL for PH were identified on chromosome 1DS and 7D (Table 6; Fig 3). The QTL peak of first one was located in the interval of *AX-94812958 and AX-110910133* in multi-environments, and physical position was located on the genomic interval of 8.97 - 21.51 Mb according to sequence assembly of *Ae. tauschii* AL8/78[27]. Under the five environmental conditions, this QTL explained 6.91 - 9.17 % of phenotypic variation (Table 6). And the QTL allele from Syn80 increased PH of seedling, with its additive effect ranging from 1.93 to 2.92 cm (Table 6). The second QTL was located in the interval of *AX-109917900-AX-110605376* with its physical interval of 324.36 -557.58 Mb in *Ae. tauschii* AL8/78. *QPh.saas-7D* explained an average PVE of 16.12 % across

multi-environments. Seedling height on the QTL allele from the parent Syn80 increased more than 5 cm in the trial of 2019GHS (Table 6). For TN, two QTL *QTn.saas-1DS* and *QTn.saas-7D* were detected under all five environmental conditions (Table 6; Fig 3). Their intervals were in accordance with the PH QTL on chromosome 1DS and 7D, respectively (Table 6; Fig 3). The PVE of *QTn.saas-1DS* ranged from 6.32 % to 19.55 % with an average of 15.34 %, and could increase about 2 tillers by allele from Syn80 in the trial of 2018GHS (Table 6).

Fig 3. QTL for PH, TN, SFW and SDW detected on 1D and 7D in five different trials.

Table 6. QTL for PH, TN, SFW and SDW in the RILs.

Traits	QTL	Environments	Peak position (cM)	Marker interval	Physical interval (Mb)	LOD	PVE (%)	ADD[a]
PH	*QPh.saas-1DS*	2017GHS	1DS:34	*AX-94812958 - AX-109908110*	8.97-11.57	4.87	9.17	-1.93
		2018GHS	1DS:34	*AX-94812958 - AX-109908110*	8.97-11.57	4.49	8.15	-2.58
		2019GHS	1DS:34	*AX-94812958 - AX-109908110*	8.97-11.57	4.86	6.91	-2.31
		2017CSS	1DS:40	*AX-94812958 - AX-110910133*	8.97-21.51	2.90	7.71	-2.92
		2018CSS	1DS:39	*AX-94812958 - AX-110910133*	8.97-21.51	2.90	7.68	-2.60
	QPh.saas-7D	2017GHS	7D:90	*AX-109917900 - AX-110605376*	324.36-557.58	7.81	14.64	-2.51
		2018GHS	7D:91	*X-109937582 - AX-110605376*	549.19-557.58	6.87	12.86	-3.33
		2019GHS	7D:91	*X-109937582 - AX-110605376*	549.19-557.58	20.16	34.33	-5.35
		2017CSS	7D:90	*AX-109917900 - AX-110605376*	324.36-557.58	4.08	9.00	-2.75
		2018CSS	7D:91	*X-109937582 - AX-110605376*	549.19-557.58	5.98	9.77	-3.31
TN	*QTn.saas-1DS*	2017GHS	1DS:33	*AX-94812958 - AX-109908110*	8.97-11.57	3.36	6.32	-0.73
		2018GHS	1DS:37	*AX-94812958 - AX-110910133*	8.97-21.51	8.82	16.34	-1.82
		2019GHS	1DS:34	*AX-94812958 - AX-109908110*	8.97-11.57	12.39	15.54	-1.54
		2017CSS	1DS:38	*AX-94812958 - AX-110910133*	8.97-21.51	10.26	19.55	-1.32
		2018CSS	1DS:35	*AX-94812958 - AX-110910133*	8.97-21.51	12.93	18.93	-1.42
	QTn.saas-7D	2017GHS	7D:91	*X-109937582 - AX-110605376*	549.19-557.58	8.38	16.68	-1.24
		2018GHS	7D:91	*X-109937582 - AX-110605376*	549.19-557.58	12.29	18.88	-2.12
		2019GHS	7D:91	*X-109937582 - AX-110605376*	549.19-557.58	26.15	38.25	-2.52
		2017CSS	7D:91	*X-109937582 - AX-110605376*	549.19-557.58	13.51	19.27	-1.46
		2018CSS	7D:91	*X-109937582 - AX-110605376*	549.19-557.58	19.48	28.66	-1.86
SFW	*QSfw.saas-1DS*	2017GHS	1DS:33	*AX-94812958 - AX-109908110*	8.97-11.57	3.70	7.51	-8.98
		2018GHS	1DS:34	*AX-94812958 - AX-109908110*	8.97-11.57	6.40	11.31	-11.17
		2019GHS	1DS:36	*AX-94812958 - AX-110910133*	8.97-21.51	7.23	12.63	-10.11
		2017CSS	1DS:42	*AX-94812958 - AX-110910133*	8.97-21.51	2.96	8.03	-8.26
		2018CSS	1DS:36	*AX-94812958 - AX-110910133*	8.97-21.51	4.27	8.71	-10.30
	QSfw.saas-7D	2017GHS	7D:91	*AX-109937582 - AX-110605376*	549.19-557.58	4.83	9.13	-10.13
		2018GHS	7D:91	*AX-109937582 - AX-110605376*	549.19-557.58	9.07	16.25	-13.70

		2019GHS	7D: 91	*AX-109937582 - AX-110605376*	549.19-557.58	15.97	26.61	-15.88
		2017CSS	7D: 91	*AX-109937582 - AX-110605376*	549.19-557.58	6.03	10.17	-10.93
		2018CSS	7D:91	*AX-109937582 - AX-110605376*	549.19-557.58	7.66	14.08	-14.22
SDW	*QSdw.saas-1DS*	2017GHS	1DS:40	*AX-94812958 - AX-110910133*	8.97-21.51	4.35	10.59	-1.39
		2018GHS	1DS:34	*AX-94812958 - AX-109908110*	8.97-11.57	6.93	12.28	-1.53
		2019GHS	1DS:36	*AX-94812958 - AX-110910133*	8.97-21.51	6.98	12.84	-1.40
		2017CSS	1DS:40	*AX-94812958 - AX-110910133*	8.97-21.51	2.73	7.24	-1.02
		2018CSS	1DS:35	*AX-94812958 - AX-110910133*	8.97-21.51	3.93	7.79	-1.28
	QSdw.saas-7D	2017GHS	7D:89	*AX-109917900 - AX-110605376*	324.36-557.58	4.40	6.53	-1.22
		2018GHS	7D:91	*AX-109937582 - AX-110605376*	549.19-557.58	8.24	14.81	-1.71
		2019GHS	7D:91	*AX-109937582 - AX-110605376*	549.19-557.58	13.10	22.20	-2.00
		2017CSS	7D:91	*AX-109937582 - AX-110605376*	549.19-557.58	5.77	10.09	-1.38
		2018CSS	7D:91	*AX-109937582 - AX-110605376*	549.19-557.58	6.88	13.24	-1.76

[a]Additive effect. Positive and negative, Syn79 and Syn80 allele produced larger value, respectively.

SDW is positively related to SFW at seedling. In this study, we detected two QTL for both SFW and SDW on chromosome 1DS and 7D (Table 6; Fig 3). And the QTL intervals for SFW and SDW were in accordance with the QTL intervals for both PH and TN. Since higher plant height and more tiller number per plant resulted in larger SFW and SDW, it suggested that they may be the same QTLs. The average PVEs for *QSfw.saas-1DS* and *QSdw.saas-1DS* were 9.64 % and 10.15%, respectively. And the QTL allele from Syn80 increased the SFW and SDW (Table 6). In the interval of *AX-109937582- AX-110605376* on chromosome 7D, QTL for both SFW and SDW were identified under all five environmental conditions, and the average PVE of *QSfw.saas-7D* and *QSdw.saas-7D* were 15.25 % and 13.37 %, respectively (Table 6). And the QTL alleles increasing SFW and SDW were from the parent Syn80 (Table 6).

In this study, ~~about~~ two genomic regions were identified to be associated with early biomass. They were in the interval of *AX-94812958 - AX-110910133* on chromosome 1DS and the interval of *AX-109917900 - AX-110605376* on chromosome 7D, respectively. The two genomic regions from the parent Syn80 could significantly enhance the early biomass with pleiotropic effects of increasing PH, TN, SFW and SDW.

Discussion

Greater early biomass is visual and important to breed new variety and innovative utilize crop germplasm, especially under adverse environmental conditions. Therefore, it is an important selective trait under drought stress [29-31], especially in Sichuan, where drought or seasonal drought occurred frequently in the last 70 years [32]. In this study, early biomass of

parents and RIL population showed significant phenotypic difference on PH, TN, SFW and SDW under the five different environmental conditions from 2016 to 2019. Phenotypic and QTL analysis demonstrated that early biomass related traits PH, TN, SFW and SDW were controlled by polygenes. Wheat growth habit types (spring or winter), the wheat growth progress and early biomass were affected by the combination of photoperiod and vernalization genes [33-36]. Photoperiod and vernalization genes on D genome were located on 2D and 5D [33,34]. Considering that the phenology and growing periods of two parents and RILs were consistent, it can be inferred that early biomass in this RILs was controlled by genes, which could not be related to photoperiod or vernalization genes, for no QTL detected on the chromosome 2D or 5D.

In the present study, two synthetic wheats Syn79 and Syn80 were generated from two different *Ae. tauschii* accessions crossing with the same tetraploid wheat, and the significant difference of early biomass between them was caused by their different D genome donors. *Ae. tauschii*, the D genome donor of common wheat, exhibited genetic diversity on early growth and might be a valuable species for improvement of early vigor in wheat [37]. The common wheat D genome progenitor *Ae. tauschii* showed a rapid leaf expansion rate at the seedling stage [21,37], which is beneficial for reducing evaporative losses from the soil [21]. Genetic dissection for early vigor related traits has been reported in several germplasm under different growing conditions, and QTL for early vigor related traits were distributed through almost the whole genome of wheat [21,37-41]. ter Steege et al identified 87 QTL for early growth related with 33 traits, 3.1 QTL per trait, explaining 32% of the phenotypic variance by using

population of *Ae. tauschii* RILs at seedling stage, but there was no significant QTLs for plant and shoot mass could be detected in his study, considering that the effects of QTL for the underlying growth traits counterbalanced each other[37]. However, in our study, the two chromosome fragments for SFW and SDW were detected, which simultaneously regulated PH and TN. And the favor alleles detected were from *Ae. Tauschii* and they could express stably in hexaploid genetic background. Few QTLs for biomass have been identified in the diploid population directly derived from *Ae. Tauschii*, but in a hexaploidy genetic background. The AABB genome of teraploid wheat may play an very important role in synthetic wheat of teraploid wheat and *Ae. tauschii*. The effects of genome combination between AABB and DD for genes expression need to be analysed in the ~~further~~. And it decleared that using SHW is a more effective method to transfer favor genes from *Ae. tauschii* to common wheat [6,7,9,42].

In addition, the two chromosome fragments for PH, TN, SFW and SDW were detected stably on 1DS and 7D, which were located on the genomic interval of 8.98 - 21.51 Mb and 324.36 - 557.58 Mb, respectively. *Lr42, Rmg6, Sr33, SrTA1662, LR10, Xa5, Chalk5, MHZ5, B10, Rc, BC10, EBR1* and *EBR1* located were in the interval of *AX-94812958 -AX-110910133* on 1DS of *Ae. tauschii*, and 16 QTL/genes (*Pid2, IPA1, Xa13, Hd18, GW8, Xa27-Xa27-IRBB27, qUVR-10, Yr33, Dn2, Ehd3, Nud, OsABCG15, MOC1, Lks2, TaD27* and QTls for antixenosis were in the interval of *AX-109917900 -AX-110605376* on 7D 0. And among these reported genes, no one except for *TaD27* associated to tiller number in

hexaploidy has been found to be related to early vigor sa far, indicating that QTLs for early biomass on 1DS in this population were new genes located in this study.

Segregation distortion is a common phenomenon among many plants 0. In the present study, 54 of 153 SNP loci showed segregation distortion in RILs, and 50 makers were skewed to Syn80 and 4 biased to Syn79. Segregation distortion loci accounted for 35.29 % of the total polymorphic loci, and 92.59 % loci preferred female parent Syn80, only 7.41 % came from male parent Syn79. At the same time, we found that Syn80 had stronger seedling vigor than one of Syn79. Therefore, the early vigor providing high survival ratio of the RILs containing Syn80 loci was higher than that of the RILs containing the Syn79 loci. The proportion of segregation distortion was high in RILs. Xu et al found the similar phenomenon, which was the purer population and higher separation ratio 0. In the present study, three genomic regions were detected to be Syn80-biased on chromosome 1D, 2D and 7D (Table 5), which were involved with the QTL intervals for early biomass. And the center of segregation bias on chromosome 1DS was located in the interval of *AX-110090502-AX-109911195* with genetic location from 8.26 cM to 12.44 cM, as 96.4 % of the progeny shared the same genotype with the parent Syn80 at the SNP site of *AX-110090502* and 97.9 % for *AX-109911195*. On chromosome 2D, the segregation bias region was framed from *AX-108911375* to *AX-110935958* across about 20 cM. And on 7D, the center of segregation bias was located in the interval of *AX-110271371* to *AX-94807766*. The center of segregation bias on 1DS was about 25 cM away from the detected QTL peaks for early growth-related traits, and the center of segregation bias on 7D was just located in the interval of QTL peaks

detected on this chromosome. Up to now, many factors may cause segregation distortion, which may be genetic factor such as reproductive isolation, or incoordination between cytoplasm and nucleus, or hybrid necrosis and so on 0, and it may be due to natural or artificial selection 0. In most cases, segregation is controlled by reproductive isolation factors such as gametophyte genes on the nucleus or sterility genes [48-51]. As several types of hybrid abnormalities including hybrid necrosis were reported in the process of synthetic wheat production 0. Usually, this abnormal growth phenotypes are often classified into hybrid necrosis (Type II and III), hybrid chlorosis and severe growth abortion 0]. And two genes derived from *Ae.tauschii* related to type II and III necrosis symptoms have been mapped 0. The gene *Nec1* of type III necrosis was on chromosome 7DS 0, while the gene *Nec2* of type II necrosis was on chromosome 2DL 0. The location of *Nec2* and *Nec1* were closed to the segregation bias region on chromosome 2D and the segregation bias center on chromosome 7D. And the reason caused the segregation bias to Syn80 in these loci was that Syn79 may carried the *Nec2* and *Nec1* alleles for hybrid necrosis. Thus, the situation of segregation bias would spread from the location of *Nec2* or *Nec1* across the QTL regions in this population. Segregation distortion regions may be related to certain genes, the gene location of the target trait can be preliminarily determined according to segregation distortion region of the genetic map and the phenotypic data. However, no strong evidence showed the early biomass QTL was caused by segregation bias to Syn80.

In the present study, 3480 SNP markers were used on the D genome, and only 153 polymorphism markers were detected between parents, the percentage polymorphic loci was

4.40%. Comparing to the genetic diversity of *Ae. taushcii* and wheat cultivars reported by previous authors [56-58], one of Syn79 and Syn80 was low on D gennome. It has been widely accepted that *Ae. tauschii* ssp. *strangulata* is the D genome donor of hexaploid wheat [56,59-63]. *Ae. tauschii* was classed to two groups, lineage 1 and lineage 2 [56,64]. Lineage 1 is broadly related to *Ae. tauschii ssp. tauschii* and lineage 2 is broadly related to *Ae. tauschii ssp. strangulata*. The Infinium SNP array for D genome was developed mainly according to the SNP polymorphism between *Ae. tauschii* ssp. tauschii and *Ae. tauschii* ssp. strangulata. Therefore, the D genome donors (AT333 and AT428) of synthetic hexaploid wheat Syn79 and Syn80 may belong to the same group (Lineage 1 or Lineage 2), and their genetic relationship is very close. Although the polymorphic loci of D genome between Syn79 and Syn80 was low, two genome regions on 1DS and 7D for four early biomass related traits were still detected under five different environments. It provided a basis for further fine mapping and candidate gene analysis of a few QTL of early biomass related traits. On the other hand, each of synthetic wheat Syn79 and Syn80 having newly genes from teraploid wheat and *Ae. tauschii*, is potential resources to diversify the genetic bottleneck in wheat breeding program.

Conclusion

By using a set of recombinant inbred lines derived from two synthetic hexaploid wheats (Syn79 and Syn80) re-synthetised from the same tetraploid wheat DOY1 and two different *Ae. tauschii* accessions (AT333 and AT428), two genomic regions on 1DS and 7D were

detected to be associated with early biomass, with pleiotropic effects on PH, TN, SFW and SDW. The QTL alleles from Syn80 enhanced the early biomass with increasing PH, TN, SFW and SDW, and originated from the Ae.tauschii AT428, which could express stably in a hexaploid background. The framed SNP markers could be used for wheat improvement.

Acknowledgments

We thank that the International Maize and Wheat Improvement Center provided synthetic hexaploid wheat parents (Syn79 and Syn80). National Natural Science Foundation of China and Department of Science and Technology of Sichuan Province supported our work. And we thank that our colleagues and staffs on Guang-Han Station and Cang-shan Sation of Sichuan Academy of Agricultural Sciences helped us with our work, too.

REFERENCES

1. Kihara H. Discovery of the DD-analyser, one of the ancestors of *Triticum vulgare.* Agric Hortic. 1944;19:889-890.

2. McFadden ES, Sears ER. The origin of *Triticum spelta* and its free-threshing hexaploid relatives. J Hered. 1946;37:107-116.

3. Bultynck L, ter Steege MW, Schortemeyer M, Poot P, Lambers H. From individual leaf elongation to whole shoot leaf area expansion;a comparison of three *Aegilops* and two *Triticum* species. Ann Bot (Lond). 2004;94:99–108.

4. Luo MC, Yang ZL, You FM, Kawahara T, Waines JG, Dvorak J. The structure of wild and domesticated emmer wheat populations, gene flow between them, and the site of emmer domestication. Theor Appl Genet. 2007;6:947–959.

5. Dvorak J, Luo MC, Yang ZL, Zhang HB. The structure of the *Aegilops tauschii* genepool and the evolution of hexaploid wheat. Theor Appl Genet. 1998;4:657–670.

6. Mujeeb-Kazi A, Rosas V, Roldan S. Conservation of the genetic variation of *Triticum tauschii (Coss.) Schmalh*. (*Aegilops squarrosa* auct. non. L) in synthetic hexaploid wheats (*T. turgidum* L. s. lat × *T. tauschii*; 2n = 6x = 42, AABBDD) and its potential utilization for wheat improvement. Genet Resour Crop. Ev. 1996;43:129–134.

7. Das MK, Bai GH, Mujeeb-Kazi A, Rajaram S. Genetic diversity among synthetic hexaploid wheat accessins (*Triticum aestivum*) with resistance to several fungal diseases. Genet. Resour Crop Ev. 2016;8:1285-1296.

8. Rana RM, Bilal M, Rehman SU, Iqbal F, Shah MKN. Synthetic wheat: a new hope for the hungry world. Asian J Agric Bio. 2013;1:91-94.

9. Yang WY, Liu DC, Li J, Zhang LQ, Wei HT, Hu XR, et al. Synthetic hexaploid wheat and its utilization for wheat genetic improvement in China. J Gene Genomics.2009;9:539-546.

10. Ren Q, Liu HJ, Zhang ZY, Feng J, Xu SC, Pu ZJ, et al. Characterization and molecular mapping of a stripe rust resistance gene in synthetic wheat C1110. J Integ. Agr. 2012;4:521-527.

11. Mulki MA, Jighly A, Ye G, Emebiri LC, Moody D, Ansari O, et al. Association mapping for soilborne pathogen resistance in synthetic hexaploid wheat. Mol Breed. 2013;2:299-311.

12. Bouhssini ME, Ogbonnaya FC, Chen M, Lhaloui S, Rihawi F, Dabbous A. Sources of resistance in primary synthetic hexaploid wheat (*Triticum aestivum* L.) to insect pests: *Hessian fly*, Russian wheat aphid and Sunn pest in the fertile crescent. Genet Resour Crop Ev. 2013;2:621-627.

13. Van Ginkel M, Ogbonnaya F, Imtiaz M, Ramage C, Borgognone MG, Dreccer F, et al. Molecular breeding for salt tolerance, pre-harvest sprouting resistance and disease resistance using synthetic hexaploid wheats, genetic transformation, and associated molecular markers. Wheat Production in Stressed Environments. 2007;12:383-385.

14. Li AL, Liu DC, Yang WY, Kishii M, Mao L. Synthetic hexaploid wheat: yesterday, today, and tomorrow. Engineering. 2018;4:552-558.

15. Yang WY, Yen C, Yang JL, Zheng YL, Liu DC. Evaluation of shape *Aegilops tauschii* Coss for resistance to physiological strains *CYR30* and *CYR31* of wheat stripe rust in China. Genet Resour Crop Ev. 1998;45:395–398.

16. Li CS, Wu XL, Tang YL, Yang WY, Wu YQ, Wu C, et al. Quality of major wheat cultivars grown in Sichuan province in recent decade. Acta Agron Sin. 2016;6:803-812 (in Chinese with English abstract).

17. Ni ZF, Zhang YR, Liang RQ, Liu GT, Sun QX. Genetic diversity of D-genome revealed by SSR markers in synthesized hexaploid wheat Introduced from CIMMYT. Acta Genetica Sinica. 2002;6:542-548 (in Chinese with English abstract).

18. Tang YL, Yang WY, Wei HT, Li CS, Li J. Opportunities for breaking the barriers of wheat yield using synthetic hexaploid wheats. Acta Scientiarum Naturalium Universitatis Sunyatseni. 2010;3:3465-3476 (in Chinese with English abstract).

19. Li J, Wan HS, Yang WY. Synthetic hexaploid wheat enhances variation and adaptive evolution of bread wheat in breeding processes. Journal of Systematics and Evolution 2014;52:735–742.

20. Wan HS, Yang YM, Li J, Zhang ZF, Yang WY. Mapping of a major QTL for hairy leaf sheath introgressed from *Aegilops tauschii* and its association with enhanced grain yield in bread wheat. Euphytica. 2015;205:275-285.

21. Li J, Wan HS, Wei HT, Wang Q, Zhou YH, Yang WY. QTL mapping for early vigor related traits in an elite wheat-breeding parent Chuanmai 42 derived from synthetic hexaploid wheat. Pak J Agri Sci. 2017;55(1):33-45.

22. Ryan PR, Liao M, Delhaize E, Rebetzke GJ, Weligama C, Spielmeyer W et al. Early vigour improves phosphate uptake in wheat. J Exp Bot. 2015;66:7089–7100.

23. Guo Y, Kong FM, Xu YF, Zhao Y, Liang X, Wang YY, et al. QTL mapping for seedling traits in wheat grown under varying concentrations of N, P and K nutrients, Theor Appl Genet. 2012;124: 851-865.

24. Yang YM, Yang WY, Wan HS, Zhang J, Li J, Lei JR, et al. Difference in growth and nutrient accumulation of synthetic wheat with different D genome under nitrogen, phosphorus and potassium stresses. J of Plant Nutrition and Fertilizer. 2015;5:1123-1131(in Chinese with English abstract).

25. Meng L, Li HH, Zhang LY, Wang JK. QTL IciMapping: integrated software for genetic linkage map construction and quantitative trait locus mapping in biparental populations. Crop J. 2015;3:269-283.

26. Li HH, Ye GY, Wang JK. A modified algorithm for the improvement of composite interval mapping. Genetics. 2007;175:361-374.

27. Luo MC, Gu YQ, Puiu D, Wang H, Twardziok SO, Deal KR, et al. Genome sequence of the progenitor of the wheat D genome *Aegilops tauschii*. Nature. 2017;551:498-502.

28. Ayalew H, Liu H, Liu CJ, Yan GJ. Identification of early vigor QTLs and QTL by environment interactions in wheat (*Triticum eastivum* L.). Plant Mol Biol Rep. 2018;36:399-405.

29. Passioura JB. Drought and drought tolerance. Plant Growth Regul. 1996;20:79-83.

30. Rebetzke GJ, Bruce SE, Kirkegaard JA. Longer coleoptiles improve emergence through crop residues to increase seedling number and biomass in wheat (*Triticum aestivum* L.). Plant Soil. 2005;272:87-100.

31. Botwright TL, Condon AG, Rebetzke GJ, Richards RA. Field evaluation of early vigour for genetic improvement of grain yield in wheat. Aust J Agric Res. 2002;53:1137-1145.

32. Deng SH, Luo XB. Features, prevention and remedy of droughts in Sichuan Since 1949. Journal of Sichuan Normal University (Social Sciences Edition). 2005;3:125-132(in Chinese with English abstract).

33. Yoshida T, Nishida H, Zhu J, Nitcher R, Distelfeld A, Akashi Y, et al. *Vrn-D4* is a vernalization gene located on the centromeric region of chromosome 5D in hexaploid wheat. Theor Appl Genet. 2010;120:543-552.

34. Beales J, Turner A, Griffiths S, Snape JW, Laurie DA. A Pseudo-Response Regulator is misexpressed in the photoperiod insensitive *Ppd-D1a* mutant of wheat (*Triticum aestivum* L.). Theor Appl Genet. 2007;115:721-733.

35. Gororo NN, Flood RG, Eastwood RF, Eagles HA. Photoperiod and Vernalization Responses in *Triticum turgidum* x *T. tauschii* Synthetic Hexaploid Wheats. Annals of Botany. 2001;88:947–952.

36. Dubcovsky J, Loukoianov A, Fu DL, Valarik M, Sanchez A and Yan LL. Effect of photoperiod on the regulation of wheat vernalization genes *VRN1* and *VRN2*. Plant Molecular Biology. 2006;60:469–480.

37. ter Steege MW, den Ouden FM, Lambers H, Stam P, Peeters AJM. Genetic and physiological architecture of early vigor in *Aegilops tauschii*, the D-genome donor of hexaploid wheat. a quantitative trait loci analysis. Plant Physiol. 2005;139:1078–1094.

38. Li J, Wan HS, Wei HT, Wang Q, Zhou YH, Yang WY. QTL mapping for early vigor related traits in an elite wheat-breeding parent Chuanmai 42 derived from synthetic hexaploid wheat. Pak J Agri Sci. 2017;55:33-45.

39. Su JY, Zheng Q, Li HW, Li B, Jing RL, Tong YP, Li ZS. Detection of QTLs for phosphorus use efficiency in relation to agronomic performance of wheat grown under phosphorus sufficient and limited conditions. Plant Sci. 2009;176:824–836.

40. Genc Y, Oldach K, Verbyla AP, Lott G, Hassan M, Tester M, et al. Sodium exclusion QTL associated with improved seedling growth in bread wheat under salinity stress. Theor Appl Genet. 2010;121:877-894.

41. Guo Y, Kong FM, Xu YF, Zhao Y, Liang X, Wang YY, et al. QTL mapping for seedling traits in wheat grown under varying concentrations of N, P and K nutrients. Theor Appl Genet. 2012;124:851-865.

42. Li AL, Liu DC, Wu J, Zhao XB, Hao M, Geng SF, et al. mRNA and small RNA transcriptomes reveal insights into dynamic homoeolog regulation of allopolyploid heterosis in nascent hexaploid wheat. Plant Cell. 2014;26:1878–1900.

43. Zhao GY, Zou C, Li K, Wang K, Li TB, Gao LF, et al. The *Aegilops tauschii* genome reveals multiple impacts of transposons. Nature Plants. 2017;3:946-955.

44. Takumi S, Motomura Y, Iehisa JCM, Kobayashi F. Segregation distortion caused by weak hybrid necrosis in recombinant inbred lines of common wheat. Genetica. 2013;141:463.

45. Xu Y, Zhu L, Xiao J, Huang N, McCouch SR. Chromosomal regions associated with segregation distortion of molecular markers in F_2 back-cross double haploid and recombinant inbred populations in rice *(Oryza sativ*a L). Mol Gen Genet. 1997;253:535-545.

46. Goloenko IM, Davydenko OG, Shimkevich AM. Segregation distortion of marker nuclear genes in alloplasmic and isoplasmic lines of barley. Russ J Genet. 2002;38:791-795.

47. Wang YJ, Wu XL, Yu DY, Zhang YM, Chen SY, Gai JY. Method of evaluation and ajustment of recombinant inbred line population and its application to the soybean RIL population NJRIKY. Acta Agronomica Sinica. 2004;30:413-418 (in Chinese with English abstract).

48. Zhao B, Deng QM, Zhang QJ, Li JQ, Ye SP, Liang YS, et al.. Analysis of segregation distortion of molecular markers in F_2 population of rice. Acta Genetica Sinica. 2006;33:449-457.

49. He P, Li JZ, Zheng XW, Shen LS, Lu MF, Chen Y, et al. Comparison of molecular linkage maps and agromomic trait loci between DH and RIL population derived from the same rice cross. Crop Science Society of America. 2001;41:1240-1246.

50. Harushima Y, Nakagahra M, Yano M, Sasaki T, Kurata N. Diverse variation of reproductive barriers in three intraspecific rice crosses. Genetics. 2002;160:313-322.

51. Harushima Y, Kurata N, Yano M, Nagamura Y, Sasaki T, Minobe Y,et al. Detection of segregation distortions in an indica-japonica rice cross using a high-resolution molecular map. Theor Appl Genet. 1996;92:145-150.

52. Nishikawa K. Hybrid lethality in crosses between Emmer wheats and *Aegilops squarrosa,* II. Synthesized 6x wheatis employed as test varieties. Jpn J Genet. 1962;37:227–236.

53. Matsuoka Y, Takumi S, Kawahara T. Natural variation for fertile triploid F_1 hybrid formation in allohexaploid wheat speciation. Theor Appl Genet. 2007;115:509–518.

54. Mizuno N, Hosogi N, Park P, Takumi S. Hypersensitive response-like reaction is associated with hybrid necrosis in interspecific crosses between tetraploid wheat and *Aegilops tauschii* Coss. PloS One. 2010;5(6):e11326. https://doi.org/10.137/journal.pone.0011326.

55. Ryusuke M, Julio CMI and Shigeo T. Application of real-time PCR-based SNP detection for mapping of *Net2*, a causal D-genome gene for hybrid necrosis in interspecific crosses between tetraploid wheat and *Aegilops tauschii*. Genes Genet Syst. 2012;87:137–143.

56. Wang JR, Luo MC, Chen ZX, You FM, Wei YM, Zheng YL, et al. *Aegilops tauschii* single nucleotide polymorphisms shed light on the origins of wheat D–genome genetic diversity and pinpoint the geographic origin of hexaploid wheat. New Phytol. 2013;198:925–937.

57. Cao TJ, Xie TZ, Wu QH, Chen YX, Wang ZZ, Zhao H, et al. Genetic diversity of registered wheat varieties in Henan province based on pedigree and single-nucleotide

polymorphism. Acta Agronomica Sinica. 2015;41:197-206 (in Chinese with English abstract).

58. Liu YK, Zhu ZW, Chen L, Zou J, Tong HW, Zhu G, et al. Revealing the genetic diversity of wheat varieties (lines) in China based on SNP markers. Acta Agronomica Sinica. 2019;http://kns.cnki.net/kcms/detail/11.1809.S.20190910.1511.012.html (in Chinese with English abstract).

59. Nishikawa K. Alpha-amylase isozymes and phylogeny of hexaploid wheat. In: Sears ER, Sears LMS, eds. 4th International Wheat Genetics Symposium. Columbia, MO, USA: Missouri Agr. Exp. Sta. 1973; 851–855.

60. Nakai Y. Isozyme variation in *Aegilops* and *Triticum*, IV. The origin of the common wheats revealed from the study on esterase isozymes in synthesized hexaploid wheats. Japan Journal of Genetics. 1979;54:175–189.

61. Lubbers EL, Gill KS, Cox TS, Gill BS. Variation of molecular markers among geographically diverse accessions of *Triticum tauschii*. Genome. 1991;34:354–361.

62. Dvorak J, Luo MC, Yang ZL. Restriction fragment length polymorphism and divergence in the genomic regions of high and low recombination in self-fertilizing and cross-fertilizing *Aegilops* species. Genetics. 1998;148:423–434.

63. Dvorak J, Deal KR, Luo MC, You FM, von Borstel K, Dehghani H. The origin of spelt and free-threshing hexaploid wheat. Journal of Heredity. 2012;103: 426–441.

64. Mizuno N, Yamasaki M, Matsuoka Y, Kawahara T, Takumi S. Population structure of wild wheat D-genome progenitor *Aegilops tauschii* Coss.: implications for intraspecific

lineage diversification and evolution of common wheat. Molecular Ecology. 2010;19:999–1013.

Figure
Click here to download Figure Fig 1..tiff

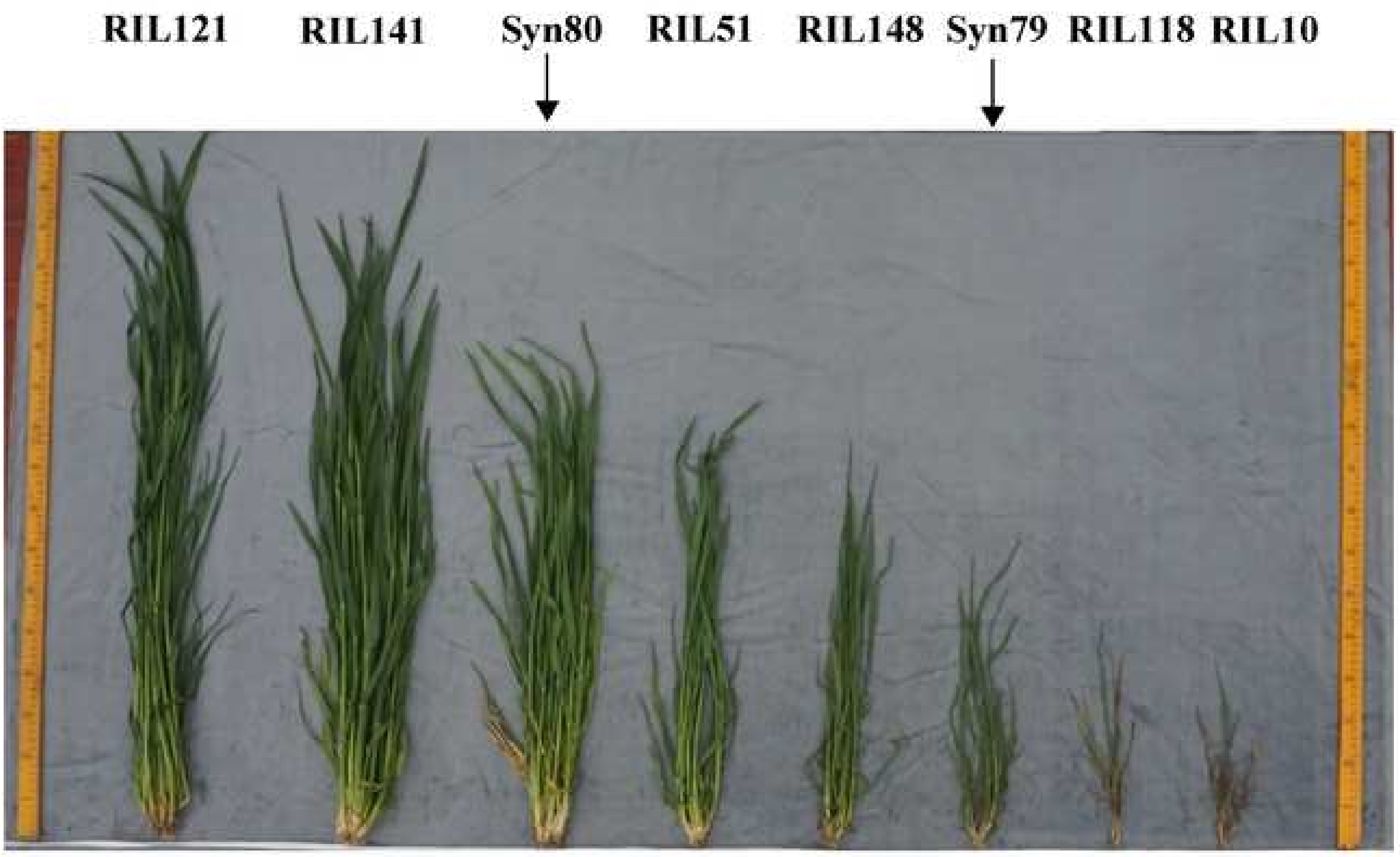

Figure

[Click here to download Figure Fig 2..tif]

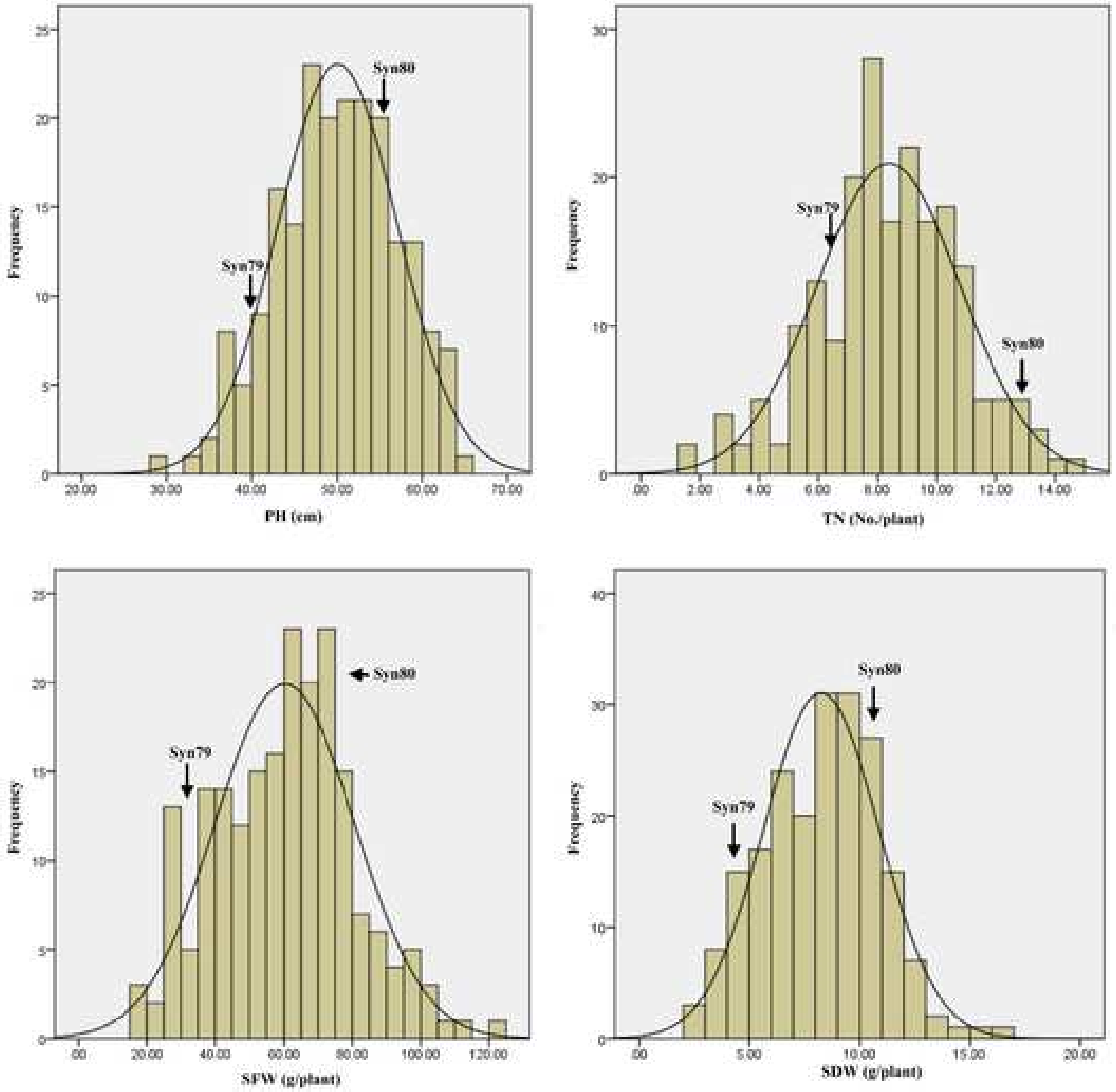

Figure

Click here to download Figure Fig 3..tif

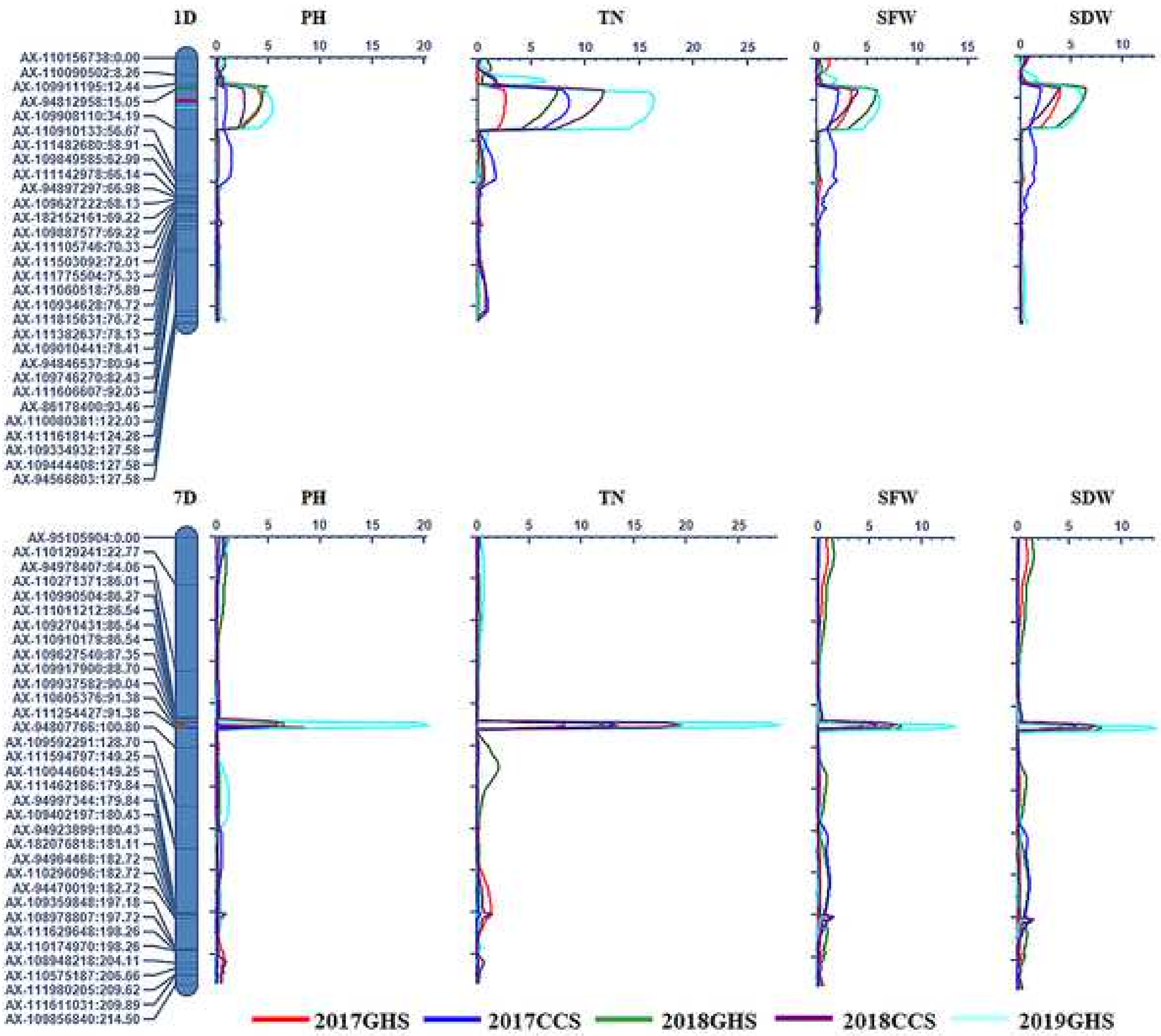

Supplement: Attachment — Submitted filename: PONE-D-20-04315_reviewer.pdf [file pone.0234882.s004.pdf]
