# Supplementary material for: Mapping QTLs for enhancing early biomass derived from Aegilops tauschii in synthetic hexaploid wheat
Source: PLoS One. 2020 Jun 25;15(6):e0234882. doi: 10.1371/journal.pone.0234882 (PMC7316292; doi:10.1371/journal.pone.0234882)
Supplement: Attachment — Submitted filename: PONE-D-20-04315_reviewer annotated.docx [file pone.0234882.s005.docx]

**PLOS ONE**

**Mapping QTL for enhancing early biomass derived from Aegilops tauschii in synthetic hexaploid wheat**

--Manuscript Draft--

| **Manuscript Number:** | PONE-D-20-04315 |
| --- | --- |
| **Article Type:** | Research Article |
| **Full Title:** | Mapping QTL for enhancing early biomass derived from Aegilops tauschii in synthetic hexaploid wheat |
| **Short Title:** | Mapping QTL in synthetic hexaploid wheat |
| **Corresponding Author:** | Wuyun Yang  Crop Research Institute Chengdu, CHINA |
| **Keywords:** |  |
| **Abstract:** | Stronger early vigor plays a crucial role in wheat improvement by enhancing resource utilization efficiency. Synthetic hexaploid wheat (SHW) combining the elite genes of tetraploid wheat and Aegilops tauschii was widely applicated in wheat genetic improvement for its abundant genetic variation. The two SHWs Syn79 and Syn80 derived from the crosses of the same tetraploid wheat DOY1 with two different Ae. tauschii AT333 and AT428, respectively. And AT428 possessed better early vigor traits than AT333. To dissect their genetic basis on D genome, 203 recombinant inbred lines (RILs) derived from the cross Syn79 x Syn80 were developed and used to detect quantitative trait loci (QTL) for four early biomass related traits, including plant height (PH), tiller number (TN), shoot fresh weight (SFW) and shoot dry weight (SDW) per plant under five different environments. With the help of SNP markers, two genome regions on 1DS and 7D were detected stably to be association with early biomass, and exhibited pleiotropic effects on PH, TN, SFW and SDW. Four stable QTL for  QPh.saas-1DS , QTn.saas-1DS , QSfw.saas-1DS and QSdw.saas-1DS were clustered in the region of 1DS from AX-94812958 to AX-110910133 , and explained 7.92, 15.34, 9.64 and 10.15% of phenotypic variation on average, respectively.  QPh.saas-7D , QTn.saas-7D , QSfw.saas-7D and QSdw.saas-7D were mapped in the interval of AX-109917900 - AX-110605376 on 7D, explaining the 16.12, 24.35,  15.25 and 13.37% of phenotypic variation on average, respectively. Moreover, the genomic regions on 1DS and 7D enhanced the early biomass by QTL alleles of the parent Syn80, which was from Ae. tauschii AT428. These findings suggest that the QTL enhancing early biomass from Ae. tauschii can express stably in hexaploid background at jointing stage and be used for wheat improvement. |
| **Order of Authors:** | Yumin Yang |
|  | Hongshen Wan |
|  | Fan Yang |
|  | Chun Xiao |
|  | Jun Li |
|  | Meijin Ye |
|  | Chunxiu Chen |
|  | Guangmin Deng |
|  | Qin Wang |
|  | Aili Li |
|  | Long Mao |
|  | Wuyun Yang |
|  | Yonghong Zhou |

| **Additional Information:** |  |
| --- | --- |
| **Question** | **Response** |
| **Financial Disclosure**  Enter a financial disclosure statement that describes the sources of funding for the work included in this submission. Review the [submission guidelines](http://journals.plos.org/plosone/s/submission-guidelines#loc-financial-disclosure-statement) for detailed requirements. View published research articles from [*PLOS ONE*](http://journals.plos.org/plosone/) for specific examples.  This statement is required for submission and **will appear in the published article** if the submission is accepted. Please make sure it is accurate. | This work was financially supported by National Natural Science Foundation of China (Grant No. 31661143007, 31401383) and Department of Science and Technology of Sichuan Province (Grant No. 2017JY0077, 2017JY0286). |
| **Unfunded studies**  Enter: *The author(s) received no specific funding for this work.*  **Funded studies**  Enter a statement with the following details:   - Initials of the authors who received each award - Grant numbers awarded to each author - The full name of each funder - URL of each funder website - Did the sponsors or funders play any role in the study design, data collection and analysis, decision to publish, or preparation of the manuscript? - **NO** - Include this sentence at the end of your statement: *The funders had no role in study design, data collection and analysis, decision to publish, or preparation of the manuscript.* - **YES** - Specify the role(s) played. |  |
| * typeset |  |
| **Competing Interests**  Use the instructions below to enter a competing interest statement for this submission. On behalf of all authors, disclose any [competing interests](http://journals.plos.org/plosone/s/competing-interests) that could be perceived to bias this work—acknowledging all financial support and any other relevant financial or non- financial competing interests. | The authors have declared that no competing interests exist. |

| This statement **will appear in the published article** if the submission is accepted. Please make sure it is accurate. View published research articles from [*PLOS ONE*](http://journals.plos.org/plosone/) for specific examples. |  |
| --- | --- |
| **NO authors have competing interests**  Enter: *The authors have declared that no competing interests exist.*  **Authors with competing interests**  Enter competing interest details beginning with this statement:  *I have read the journal's policy and the authors of this manuscript have the following competing interests: [insert competing interests here]* |  |
| * typeset |  |
| **Ethics Statement**  Enter an ethics statement for this submission. This statement is required if the study involved:   - Human participants - Human specimens or tissue - Vertebrate animals or cephalopods - Vertebrate embryos or tissues - Field research   Write "N/A" if the submission does not require an ethics statement.  General guidance is provided below. Consult the [submission guidelines](http://journals.plos.org/plosone/s/submission-guidelines#loc-guidelines-for-specific-study-types) for detailed instructions. **Make sure that all information entered here is included in the Methods section of the manuscript.** | N/A |

| **Format for specific study types**  **Human Subject Research (involving human participants and/or tissue)**   - Give the name of the institutional review board or ethics committee that approved the study - Include the approval number and/or a statement indicating approval of this research - Indicate the form of consent obtained (written/oral) or the reason that consent was not obtained (e.g. the data were analyzed anonymously)   **Animal Research (involving vertebrate animals, embryos or tissues)**   - Provide the name of the Institutional Animal Care and Use Committee (IACUC) or other relevant ethics board that reviewed the study protocol, and indicate whether they approved this research or granted a formal waiver of ethical approval - Include an approval number if one was obtained - If the study involved *non-human primates*, add *additional details* about animal welfare and steps taken to ameliorate suffering - If anesthesia, euthanasia, or any kind of animal sacrifice is part of the study, include briefly which substances and/or methods were applied   **Field Research**  Include the following details if this study involves the collection of plant, animal, or other materials from a natural setting:   - Field permit number - Name of the institution or relevant body that granted permission |  |
| --- | --- |
|  |  |
| **Data Availability**  Authors are required to make all data underlying the findings described fully available, without restriction, and from the time of publication. PLOS allows rare exceptions to address legal and ethical concerns. See the [PLOS Data Policy](http://journals.plos.org/plosone/s/data-availability) and [FAQ](http://journals.plos.org/plosone/s/data-availability#loc-faqs-for-data-policy) for detailed information. | Yes - all data are fully available without restriction |

| A Data Availability Statement describing where the data can be found is required at submission. Your answers to this question constitute the Data Availability Statement and **will be published in the article**, if accepted. |  |
| --- | --- |
| **Important:** Stating ‘data available on request from the author’ is not sufficient. If your data are only available upon request, select ‘No’ for the first question and explain your exceptional situation in the text box. |  |
| Do the authors confirm that all data underlying the findings described in their manuscript are fully available without restriction? |  |
| **Describe where the data may be found in full sentences. If you are copying our sample text, replace any instances of XXX with the appropriate details.**   - If the data are **held or will be held in a public repository**, include URLs, accession numbers or DOIs. If this information will only be available after acceptance, indicate this by ticking the box below. For example: *All XXX files are available from the XXX database (accession number(s) XXX, XXX.*). - If the data are all contained **within the manuscript and/or Supporting Information files**, enter the following: *All relevant data are within the manuscript and its Supporting Information files.* - If neither of these applies but you are able to provide **details of access elsewhere**, with or without limitations, please do so. For example:   *Data cannot be shared publicly because of [XXX]. Data are available from the XXX Institutional Data Access / Ethics Committee (contact via XXX) for researchers who meet the criteria for access to confidential data.*  *The data underlying the results presented in the study are available from (include the name of the third party* | All relevant data are within the manuscript and its Supporting Information files. |

| *and contact information or URL).*   - This text is appropriate if the data are owned by a third party and authors do not have permission to share the data.   * typeset |  |
| --- | --- |
| Additional data availability information: |  |

Manuscript	[Click here to download Manuscript Manuscript.doc](https://www.editorialmanager.com/pone/download.aspx?id=26045929&guid=468f1186-ccf0-4e42-8fb7-b624559070bf&scheme=1)

1 **Mapping QTL for enhancing early biomass derived**
2 **from *Aegilops tauschii* in synthetic hexaploid wheat**

3

4

5 Yumin Yang1,2,4,¶, Hongshen Wan3,4,¶, Fan Yang^1^, Chun Xiao^2^, Jun Li^3,4^, Meijin Ye^1^,
6 Chunxiu Chen^2^, Guangmin Deng^2^, Qin Wang^3,4^, Aili Li^5^, Long Mao^5^, Wuyun Yang^3,4*^,
7 Yonghong Zhou^1*^ 8

9

10

11 1 Triticeae Research Institute, Sichuan Agricultural University, Chengdu, China
12 2 Soil and Fertilizer Research Institute, Sichuan Academy of Agricultural Sciences, Chengdu,
13 China
14 3 Crop Research Institute, Sichuan Academy of Agricultural Sciences, Chengdu, China
15 4 Key Laboratory of Wheat Biology and Genetic Improvement on Southwestern China
16 (Ministry of Agriculture and Rural Areas), Chengdu, China
17 5 Institute of Crop Sciences, Chinese Academy of Agricultural Sciences, Beijing, China 18

19

20 ***** Corresponding author
21 E-mail addresses: [yangwuyun@126.com](mailto:yangwuyun@126.com) (W. Yang), [zhouyhdavid@126.com](mailto:zhouyhdavid@126.com) (Y. Zhou) 22
23 ¶ These authors contributed equally to this work.

# 24 Abstract

25 Strong early vigor plays a crucial role in wheat yield improvement by enhancing resource
26 utilization efficiency. Synthetic hexaploid wheat (SHW) combining the elite genes of
27 tetraploid wheat and *Aegilops tauschii* was widely used in wheat improvement
28 for its abundant genetic variation. The two SHWs Syn79 and Syn80 were derived from crossing
29 the same tetraploid wheat DOY1 with two different *Ae. tauschii* accessions AT333 and AT428,
30 respectively. The AT428 possessed better early vigor traits than AT333. To dissect the
31 genetic basis on D genome, 203 recombinant inbred lines (RILs) derived from the cross
32 Syn79 x Syn80 were developed to detect quantitative trait loci (QTL) for four
33 biomass related traits, including plant height (PH), tiller number (TN), shoot fresh weight
34 (SFW) and shoot dry weight (SDW) per plant, under five different environments. Determined from
35 the data of SNP markers, two genome regions on 1DS and 7D were stably
36 associated with the four early biomass traits showing pleiotropic effects.
37 Four stable QTL *QPh.saas-1DS*, *QTn.saas-1DS*, *QSfw.saas-1DS* and *QSdw.saas-1DS* explaining 7.92, 15.34, 9.64 and 10.15 % of phenotypic variation, respectively,
38 were clustered in the region of 1DS from *AX-94812958* to *AX-110910133*
39 . Meanwhile, *QPh.saas-7D*,
40 *QTn.saas-7D*,	*QSfw.saas-7D*	and	*QSdw.saas-7D*	were	flanked by

41	*AX-109917900* and *AX-110605376* on 7D, explaining the 16.12, 24.35, 15.25 and 13.37 % of

42 phenotypic variation on average, respectively. Moreover, the genomic QTL regions on 1DS and
43 7D enhancing biomass in the parent Syn80 were from *Ae.*
44 *tauschii* AT428. These findings suggest that the QTLs from *Ae.*
45 *tauschii* can be expressed stably in hexaploid background at the jointing stage and be used for wheat
46 improvement.

47

# 48 Introduction

49 Common wheat (*Triticum aestivum* L., 2n = 6x = 42, AABBDD), as an important food
50 crop in the world, originated from the spontaneous hybridization of tetraploid *Triticum*
51 *turgidum* wheat (2n = 4x = 28, AABB) with diploid *Aegilops tauschii* Coss (2n = 2x = 14,
52 DD) [1,2]. It is believed that only a few accessions of the donor species were involved in the
53 evolution of common wheat, especially for the D genome donor. Consequently, the genetic
54 diversity of common wheat was significantly decreased in comparison with its donor species.
55 Due to the evolutionary bottleneck, most of the genetic variation in *Ae. tauschii* did not exist
56 in the commonly available hexaploid germplasm [3], and only 7 % of the variants observed
57 in *Ae. tauschii* were reserved in common wheat [4,5]. To enhance the transferring efficiency
58 of elite genes from *Ae. tauschii* species to common wheat, scientists created synthetic
59 hexaploid wheat (SHW) from crosses between *T. turgidum* and *Ae. tauschii* to broaden the
60 genetic variation of hexaploid wheat [6]. Over 1000 SHW lines have been produced by using
61 more than 600 *Ae. tauschii* accessions stored at the International Maize and Wheat
62 Improvement Center (CIMMYT; Mexico City, Mexico) [7]. SHWs with huge genetic
63 diversity have shown their outstanding superiority in resistance to diseases and pests,
64 tolerance to environmental stresses, and desirable quantitative traits, and been used widely in
65 common wheat breeding [8-14]. Chinese scientists have shown a high interest in CIMMYT
66 SHW since the early 1990s [15-18]. More than 200 CIMMYT SHW accessions have been
67 introduced into China in 1995 [14]. In recent years, several commercial wheat varieties have
68 been also created and released in China [9,14,19]. Furthermore, several favorable
69 introgressions from *Ae. tauschii* have been identified in synthetic derivatives [19]. A major
70 QTL on 4DL associated with leaf sheath hairiness in a synthetic derivative wheat variety
71 Chuanmai42 was detected, and its wild allele was originated from *Ae. tauschii*, which has
72 significantly increased grain weight, grain yield, and yield related characters [20].
73 Vigorous cultivars have advantages to enhance the population water-use efficiency
74 by shading the soil surface faster and reducing evaporative losses from the soil [21-23].
75 Rapid early development of leaf area and root system are associated with increased water and
76 nutrient use efficiency, high rates of light interception and biomass production resulting in drought tolerance and
77 high yield potential [22, 23]. In recent years, we have screened CIMMYT
78 SHWs for high biomass and found two SHWs (Syn79 and Syn80) derived from the same tetraploid wheat
79 (durum wheat DOY1) and two different *Ae. tauschii* accessions having significantly different
80 biomass in the whole developing stage. We attributed the significant difference of biomass between
81 the two SHWs to the different genotypes in the two D genome donors. the vegetative growth, nutrient accumulation,
82 nutrient distribution and utilization of Syn79 and Syn80 were significantly different under
83 different environments [24]. In order to evaluate the genetic impact of the different D
84 genomes on early vigor in hexaploid wheat, a population of recombinant inbred lines (RILs)
85 derived from a cross between Syn79 and Syn80 have been developed. The goal of this study
86 was to map the major QTLs associated with early biomass accumulation contributed from *Ae. tauschii* in hexaploid
87 wheat background at jointing stage for the molecular breeding of wheat yield using
88 SHWs.

# 89 Materials and methods

## 90 Plant materials

91 Two hundred and three F9 recombinant inbred lines (RILs) derived from Syn79 x Syn80
92 cross and their parents were used for QTL mapping in this study. Syn79 and Syn80 were
93 generated from durum wheat DOY1 (2n=28, AABB) crossing with *Ae. tauschii* (2n=14, DD)
94 by CIMMYT [6]. The AB genome of Syn79 and Syn80 was from the same durum donor
95 DOY1, while their D genomes were from two different *Ae. tauschii* accessions (AT333 and
96 AT428). Syn80 had stronger early vigor than Syn79 (Fig 1), which was caused by their
97 different D genome, and AT428 possessed better early vigor traits than AT333.

98

99

100

### Fig 1. Early growth of the two parents and their RILs in the jointing stage.

101 **Field trials**
102 A total of five trials for Syn79, Syn80 and 203 RILs were conducted at Guang-Han
103 Station (GHS) in 2017-2019 (2017GHS, 2018GHS, 2019GHS) and Cang-Shan Station (CSS)
104 in 2017 and 2018 (2017CSS, 2018CSS). GHS and CSS of Sichuan Academy of Agricultural
105 Sciences (SAAS) are the representative of the plain region and hilly region in Sichuan
106 province, respectively. Chemical properties of their soils of five trials were shown in Table 1.
107 The organic matter, total nitrogen and available nitrogen of soil in GHS were significantly
108 higher than those in CCS, and the total potassium of soil in CSS was more than that in GHS
109 (Table 1).
110 110

### 111 Table 1. Chemical properties of soil in different field trials.

| Trials | | pH | Organic matter | Total Total  nitrogen phosphorus | | Total potassium | Available nitrogen | Available phosphorus | Available potassium |
| --- | --- | --- | --- | --- | --- | --- | --- | --- | --- |
|  |  | | (g/kg) | (g/kg) | (g/kg) | (g/kg) | (mg/kg) | (mg/kg) | (mg/kg) |
|  | 2017GHS 6.84 | | 31.9 | 1.99 | 0.723 | 16.25 | 165 | 6.9 | 90 |
|  | 2018GHS 6.45 | | 39.7 | 2.31 | 0.860 | 18.77 | 206 | 15.8 | 105 |
|  | 2019GHS 6.71 | | 28.9 | 2.03 | 0.674 | 19.00 | 183 | 11.0 | 96 |
|  | 2017CSS 7.81 | | 9.5 | 0.77 | 0.556 | 23.81 | 47 | 3.7 | 100 |
|  | 2018CSS 8.24 | | 15.7 | 1.23 | 0.328 | 22.90 | 97 | 2.9 | 137 |
| 112 |  | |  |  |  |  |  |  |  |

113 The trials were performed in randomized complete blocks with three replicates. Each
114 plot had five 1.5 m rows spaced 0.5 m apart. At two-leaf stage, only ten evenly distributed
115 plants in each row were retained for further growth. Field management consisted of
116 commonly under-taken practices in wheat production.

## 117 Trait evaluation

118 Four early biomass related traits, plant height (PH), tiller number (TN), shoot fresh
119 weight (SFW) and shoot dry weight (SDW) per plant were investigated RILs and their
120 parents at the jointing stage. In each plot, 10 plants with whole tissues including root and
121 shoot were randomly selected to evaluate traits associated with early biomass. Sampling at
122 the jointing stage, plants at the ends of each row were disregarded to minimize within-row
123 edge effects. After sampling, the roots of the 10 plants were cut, PH, TN, SFW and SDW
124 were measured. All traits were described by the mean values of 10 plants for corresponding
125 line. The measurement for SFW was accomplished within 12 hours after sampling.

## 126 SNP Genotyping

127 A total of 50 mg of plant fresh leaves was collected from 2-week-old seedlings and
128 DNA was extracted using the NuClean Plant Genomic DNA Kit (CWBio, Beijing, China).
129 Eluted DNA was quantified using Qubit 4 Fluorometer (Life Technologies Holdings Pte Ltd,
130 Singapore) and then normalized using a 12-channel electronic pipette with a volume ranging from
131 10 to 100µL (Eppendorf, Hamburg, Germany) to obtain the concentration required for
132 genotyping.
133 The RILs and their parents Syn79 and Syn80 were executed on the Affymetrix platform
134 of Axiom Wheat Breeder’s Genotyping Array with 13947 SNP markers including 1272
135 functional markers by China Golden Marker Biotech Co Ltd (Beijing, China). The collected
136 fluorescence signal from SNP array were processed and analyzed by the functions of
137 apt-genotype-axiom for genotype calling, ps-metrics for generating various QC metrics and
138 ps-classification for classifying SNPs in the software of Affymetrix Axiom Analysis Suite
139 version 4.0.1. Among 13947 SNP markers, a total of 3480 SNPs is distributed on D genome,
140 and was used for parental polymorphism analysis.

## 141 Statistical and QTL analysis

142 Descriptive analysis, analysis of variance (ANOVA) and correlation analysis for the
143 phenotypic data were calculated using the SPSS statistical package (SPSS Inc., Chicago, IL).
144 Variation of genotypes for phenotypic traits were evaluated using mean, stansard deviation
145 (SD), coefficient of variation (CV), maximum (Max) and minimum (Min).Variance
146 components of ANOVA for all traits was calculated based the general linear model (GLM) to
147 detected the effect of genotypes, environments and genotypes × environments interaction.
148 Broad sense heritability (H^2^) was estimated with the formula: H^2^= σ^2^g/ (σ^2^g + σ^2^ge/n +
149 σ^2^e/nr), where σ^2^g is genetic variance, σ^2^ge is the variance of genotype-environment
150 interaction, σ^2^e is experiment error variance, n is the number of trials, r is the number of
151 replications.
152 The QTL IciMapping Software version 4.1 [25,26] was used for genetic linkage map
153 construction. The location of SNP marker was aligned according to the physical map of *Ae.*
154 *tauschii* AL8/78 for D genome [27]. The genetic linkage map was constructed according to
155 153 polymorphic markers between Syn79 and Syn80 (the parents), which were screened from
156 3480 SNP markers distributed on D genome. The map covered over 803.84 cM on wheat D
157 genome, with an average distance of 5.25 cM between adjacent polymorphic markers.
158 QTL analysis for the measured traits under the five different environmental conditions
159 were performed using the inclusive composite interval mapping (ICIM) option on the QTL
160 IciMapping Software version 4.1. Significant	LOD threshold was determined by	1000
161 permutations and P = 0.05. Linked QTL with the genetic distance less than 20 cM were
162 considered as one single QTL, which were named according to Ayalew et al. [28].

# 163 Results

## 164 Phenotypic analysis

165 Five different field trials were conducted at two locations over 3 years to evaluate early
166 biomass related traits of the RIL population as well as their parents Syn79 and Syn80. Syn80
167 have greater early biomass than Syn79 (Fig 1). The values of PH, TN, SFW and SDW for
168 Syn80 were significantly larger than those of Syn79 under all five environments (Table 2).
169 Independent of the differences between the two parents, in all trials there were significant
170 variations in the investigated traits of the RIL populations, with values spanning much larger
171 ranges than those defined by the parental values. And the phenotypic data was distributed
172 normally in the RILs (Fig 2). Variation of phenotypic data was tremendous in the RILs,
173 especially for SFW and SDW. And variation was determined by genotypes, environments
174 and genotypes × environments interaction. Their heritability ranged from 39.20 to 43.27 %
175 (Table 2). It suggested that those phenotypic traits were controlled by multigene, and these
176 176
177 177

genes’ phenotypic traits were significantly affected by environment .

178 178
179 179
180 180

### Fig 2. Distribution graph of the phenotypic data for PH, TN, SFW and SDW.

181 181
182 182

### Table 2. Parental values, population distribution parameters, and heritability of the investigated traits

Trait	Environment	Parents	RILs	H^2^	F-values from ANOVA

|  | Syn79 | Syn80 | Mean±SD | CV(%) | Min-Max | (%) | Environment | Genotype | Environment×genotype |
| --- | --- | --- | --- | --- | --- | --- | --- | --- | --- |
| PH 2017GHS | 31.87 | 48.64^**^ | 38.43±5.98 | 15.56 | 27.00-59.20 | 43.27 | 1368.74^**^ | 14.05^**^ | 2.02^**^ |
| (cm) 2018GHS | 34.48 | 53.81^**^ | 50.12±8.07 | 16.10 | 29.38-65.33 |  |  |  |  |
| 2019GHS | 46.11 | 63.33^**^ | 60.87±8.20 | 13.47 | 38.11-81.94 |  |  |  |  |
| 2017CSS | 42.00 | 56.33^**^ | 54.40±8.34 | 15.33 | 27.17-69.33 |  |  |  |  |
| 2018CSS | 49.67 | 65.11^**^ | 59.84±9.07 | 15.16 | 27.58-76.56 |  |  |  |  |
| TN 2017GHS | 5.60 | 12.53^**^ | 10.01±2.58 | 25.77 | 4.50-17.90 | 43.11 | 780.24^**^ | 14.32^**^ | 2.05^**^ |
| (No./plant) 2018GHS | 8.17 | 16.67^**^ | 15.05±3.92 | 26.05 | 6.00-23.00 |  |  |  |  |
| 2019GHS | 10.00 | 15.78^**^ | 14.23±3.73 | 26.21 | 5.78-21.67 |  |  |  |  |
| 2017CSS | 7.00 | 12.00^**^ | 9.22±2.58 | 27.98 | 2.00-15.00 |  |  |  |  |
| 2018CSS | 8.33 | 13.22^**^ | 11.16±3.31 | 29.66 | 2.33-18.33 |  |  |  |  |
| SFW 2017GHS | 17.09 | 73.72^**^ | 48.25±25.60 | 53.06 | 7.02-136.54 | 40.20 | 144.40^**^ | 14.05^**^ | 2.16^**^ |
| (g/plant) 2018GHS | 21.79 | 70.78^**^ | 60.92±29.91 | 49.10 | 7.30-156.94 |  |  |  |  |
| 2019GHS | 29.40 | 78.37^**^ | 64.94±29.89 | 46.03 | 10.97-169.70 |  |  |  |  |
| 2017CSS | 26.58 | 82.57^**^ | 52.69±25.50 | 48.40 | 3.59-132.55 |  |  |  |  |
| 2018CSS | 36.89 | 96.08^**^ | 75.44±39.27 | 52.05 | 4.72-158.86 |  |  |  |  |
| SDW 2017GHS | 2.85 | 10.22^**^ | 6.64±3.34 | 50.30 | 1.04-16.19 | 39.20 | 161.47^**^ | 84.08^**^ | 6.60^**^ |
| (g/plant) 2018GHS | 3.43 | 10.24^**^ | 8.35±3.58 | 42.87 | 1.04-20.54 |  |  |  |  |
| 2019GHS | 4.63 | 11.87^**^ | 8.96±4.31 | 48.10 | 1.43-17.80 |  |  |  |  |
| 2017CSS | 4.43 | 11.45^**^ | 7.25±3.28 | 45.24 | 0.46-18.13 |  |  |  |  |
| 2018CSS | 5.59 | 13.98^**^ | 10.18±4.95 | 48.62 | 0.69-20.57 |  |  |  |  |

183 * And ** indicate significant differences at P= 0.05 and 0.01, respectively.
184 Correlations among the PH, TN, SFW and SDW in different trials are presented in Table
185 3. It showed that significantly positive correlations among these traits were detected in early
186 growth stage. Their average coefficients in five trials ranged from 0.581 to 0.975. The PH
187 was significantly positively correlated with TN, and the coefficients ranged from 0.424 to
188 0.683 in different trials (Table 3). Both PH and TN showed significant positive correlations
189 with SFW and SDW, and the coefficients were higher than that between PH and TN. These
190 results suggested that greater early biomass was related with higher PH, more TN, heavier
191 SFW and SDW, and the gene related to early biomass had pleiotropic effects on all four
192 traits.
193 193
194 **Table 3. Correlation coefficients between the four traits in RILs in different trials.**

|  | TN | SFW | SDW |
| --- | --- | --- | --- |
| PH | O.673^**^ | 0.724^**^ | 0.733^**^ |
|  | 0.424^**^ | 0.364^**^ | 0.355^**^ |
|  | 0.683^**^ | 0.784^**^ | 0.808^**^ |
| TN | 0.606^**^  0.612^**^ | 0.821^**^  0.791^**^  0.708^**^ | 0.835^**^  0.791^**^  0.711^**^ |
|  |  | 0.142^*^ | 0.141^*^ |
|  |  | 0.731^**^ | 0.728^**^ |
|  |  | 0.668^**^ | 0.678^**^ |
|  |  | 0.749^**^ | 0.713^**^ |
| SFW |  |  | 0.967^**^ |
|  |  |  | 0.969^**^ |
|  |  |  | 0.978^**^ |
|  |  |  | 0.983^**^ |
|  |  |  | 0.980^**^ |

195 ^*^And** indicate significance at P=0.05 and 0.01 level, respectively. 196

## 197 Genetic map of D genome

198 In this study, we used Wheat Breeder’s Genotyping Array to genotype the A, B and D
199 genome, respectively. For AB genome, a total of 10467 SNP labels anchored on the
200 genotyping array were used to check the genotype of AB genome of RIL population and their
201 parents, which were generated from the same AB donor. And the results showed that almost
202 all SNP markers on AB genome had no polymorphism between two parents. For D genome,
203 3480 SNP labels were selected to fix on the chip by China Golden Marker Biotech Co Ltd
204 (Beijing, China). Among these scanned markers, 153 markers on D genome had
205 polymorphism between two parents, and distributed unequally on the seven chromosomes of
206 the D genome (Table 4). The number of polymorphic markers on different chromosomes
207 ranged from 8 on 3D to 34 on 7D (Table 4).
208 208

### 209 Table 4. SNP markers on the D genome.

|  | Parameter | 1D | 2D | 3D | 4D | 5D | 6D | 7D | Total |
| --- | --- | --- | --- | --- | --- | --- | --- | --- | --- |
|  | Total Makers | 370 | 634 | 536 | 265 | 550 | 428 | 697 | 3480 |
|  | Polymorphic markers | 30 | 20 | 8 | 21 | 25 | 15 | 34 | 153 |
|  | Polymorphism rate (%) | 8.11 | 3.15 | 1.49 | 7.92 | 4.55 | 3.50 | 4.88 | 4.40 |
|  | Map length (cM) | 127.58 | 69.22 | 21.35 | 145.52 | 135.72 | 89.95 | 214.50 | 803.84 |
|  | Distance between polymorphic markers | 4.25 | 3.46 | 2.67 | 6.93 | 5.43 | 6.00 | 6.31 | 5.25 |
|  | (cM) |  |  |  |  |  |  |  |  |
| 210 |  |  |  |  |  |  |  |  |  |

211 For linkage map construction, SNP markers were grouped according to their anchored
212 chromosomes of *Ae. tauschii* AL8/78 for D genome, and then aligned by nnTwoOpt method.
213 The whole genetic map covered over 803.84 cM on D genome with an average distance
214 between adjacent markers of 5.25 cM (Table 4). There were no genetic gaps with adjacent
215 marker distance more than 50 cM in each chromosome, and the average distance between
216 two adjacent markers ranged from 2.67 cM to 6.93 cM. For all the 7 chromosomes, the
217 linkage maps ranged from 21.35 cM to 214.50 cM. For the chromosome 2D and 3D, the total
218 distance of the linkage maps constructed by this population and the Wheat Breeder’s
219 Genotyping Array were 69.22 cM and 21.35 cM, respectively. Out of the genomic regions of
220 linkage maps, no polymorphic markers were detected by this SNP array.
221 Genotypic markers were tested for segregation distortion (deviation from the expected 1:1 ratio)
222 by Chi-squared tests. Among the 153 SNP loci, 54 loci showed segregation distortion in RILs
223 (Table 5). Almost all loci were biased to Syn80 with larger early biomass, which means that
224 in those loci most of the RILs progeny preferentially inherited the female parent Syn80. And
225 only four loci were male-biased (Table 5). Among those female-biased loci, the number of
226 loci on different chromosomes distributed from 1 on 3D to 18 on 7D. And three genomic
227 regions were detected to be Syn80-biased on chromosome 1D, 2D and 7D (Table 5), and
228 covered about 50, 20 and 40 cM on 1D, 2D and 7D, respectively. 229

### 230 Table 5. Segregation distortion of SNP loci in RILs

Chromosome	Syn80-biased Locus	Unbiased Locus	Syn79-biased Locus

|  | Number | Rate (%) | Number | Rate (%) | Number | Rate (%) |
| --- | --- | --- | --- | --- | --- | --- |
| 1D | 11 | 36.67 | 19 | 63.33 | 0 | 0.00 |

| 2D | 14 | 70.00 | 5 | 25.00 | 1 | 5.00 |
| --- | --- | --- | --- | --- | --- | --- |
| 3D | 1 | 12.50 | 7 | 87.50 | 0 | 0.00 |
| 4D | 2 | 9.52 | 19 | 90.48 | 0 | 0.00 |
| 5D | 3 | 12.00 | 20 | 80.00 | 2 | 8.00 |
| 6D | 1 | 6.67 | 14 | 93.33 | 0 | 0.00 |
| 7D | 18 | 52.94 | 15 | 44.12 | 1 | 2.94 |
| Total | 50 | 32.68 | 99 | 64.71 | 4 | 2.61 |

231 Underline means genetic regions with linked loci; Chi-squared test were programmed at

| 232 | P=0.05 level |
| --- | --- |
| 233 |  |
| 234 | **QTL on D genome** |
| 235 | With the linkage map constructed by 153 SNP markers on D genome, QTL for PH, TN, |
| 236 | SFW and SDW were identified under five environmental conditions using the inclusive |
| 237 | composite interval mapping (ICIM) program. |
| 238 | PH and TN are common agronomic traits in wheat, and the higher PH and TN in seeding |
| 239 | growth stage are positively correlated with enhanced water-use efficiency of the population |
| 240 | by shading the soil surface faster and reducing evaporative losses from the soil. A total of two |
| 241 | QTL for PH were identified on chromosome 1DS and 7D (Table 6; Fig 3). The QTL peak of |
| 242 | first one was located in the interval of *AX-94812958 and AX-110910133* in |
| 243 | multi-environments, and physical position was located on the genomic interval of 8.97 - |
| 244 | 21.51 Mb according to sequence assembly of *Ae. tauschii* AL8/78 [27]. Under the five |
| 245 | environmental conditions, this QTL explained 6.91 - 9.17 % of phenotypic variation (Table |
| 246 | 6). And the QTL allele from Syn80 increased PH of seedling, with its additive effect ranging |

247 from 1.93 to 2.92 cm (Table 6). The second QTL was located in the interval of
248 *AX-109917900-AX-110605376* with its physical interval of 324.36 -557.58 Mb in *Ae.*
249 *tauschii*	AL8/78.	*QPh.saas-7D*	explained	an	average	PVE	of	16.12	%	across
250 multi-environments. Seedling height on the QTL allele from the parent Syn80 increased more
251 than 5 cm in the trial of 2019GHS (Table 6). For TN, two QTL *QTn.saas-1DS* and
252 *QTn.saas-7D* were detected under all five environmental conditions (Table 6; Fig 3). Their
253 intervals were in accordance with the PH QTL on chromosome 1DS and 7D, respectively
254 (Table 6; Fig 3). The PVE of *QTn.saas-1DS* ranged from 6.32 % to 19.55 % with an average
255 of 15.34 %, and could increase about 2 tillers by allele from Syn80 in the trial of 2018GHS
256 256
257 257

(Table 6).

258 258
259 259
260 260

### Fig 3. QTL for PH, TN, SFW and SDW detected on 1D and 7D in five different trials.

261 261

### Table 6. QTL for PH, TN, SFW and SDW in the RILs.

Traits	QTL	Environments Peak position

| (cM) | | | | (Mb) | | (%) | | |
| --- | --- | --- | --- | --- | --- | --- | --- | --- |
| PH | *QPh.saas-1DS* | 2017GHS | 1DS:34 | *AX-94812958 - AX-109908110* | 8.97-11.57 | 4.87 | 9.17 | -1.93 |
|  |  | 2018GHS | 1DS:34 | *AX-94812958 - AX-109908110* | 8.97-11.57 | 4.49 | 8.15 | -2.58 |
|  |  | 2019GHS | 1DS:34 | *AX-94812958 - AX-109908110* | 8.97-11.57 | 4.86 | 6.91 | -2.31 |
|  |  | 2017CSS | 1DS:40 | *AX-94812958 - AX-110910133* | 8.97-21.51 | 2.90 | 7.71 | -2.92 |
|  |  | 2018CSS | 1DS:39 | *AX-94812958 - AX-110910133* | 8.97-21.51 | 2.90 | 7.68 | -2.60 |
|  | *QPh.saas-7D* | 2017GHS | 7D:90 | *AX-109917900 - AX-110605376* | 324.36-557.58 | 7.81 | 14.64 | -2.51 |
|  |  | 2018GHS | 7D:91 | *X-109937582 - AX-110605376* | 549.19-557.58 | 6.87 | 12.86 | -3.33 |
|  |  | 2019GHS | 7D:91 | *X-109937582 - AX-110605376* | 549.19-557.58 | 20.16 | 34.33 | -5.35 |
|  |  | 2017CSS | 7D:90 | *AX-109917900 - AX-110605376* | 324.36-557.58 | 4.08 | 9.00 | -2.75 |
|  |  | 2018CSS | 7D:91 | *X-109937582 - AX-110605376* | 549.19-557.58 | 5.98 | 9.77 | -3.31 |
| TN | *QTn.saas-1DS* | 2017GHS | 1DS:33 | *AX-94812958 - AX-109908110* | 8.97-11.57 | 3.36 | 6.32 | -0.73 |
|  |  | 2018GHS | 1DS:37 | *AX-94812958 - AX-110910133* | 8.97-21.51 | 8.82 | 16.34 | -1.82 |
|  |  | 2019GHS | 1DS:34 | *AX-94812958 - AX-109908110* | 8.97-11.57 | 12.39 | 15.54 | -1.54 |
|  |  | 2017CSS | 1DS:38 | *AX-94812958 - AX-110910133* | 8.97-21.51 | 10.26 | 19.55 | -1.32 |
|  |  | 2018CSS | 1DS:35 | *AX-94812958 - AX-110910133* | 8.97-21.51 | 12.93 | 18.93 | -1.42 |
|  | *QTn.saas-7D* | 2017GHS | 7D:91 | *X-109937582 - AX-110605376* | 549.19-557.58 | 8.38 | 16.68 | -1.24 |
|  |  | 2018GHS | 7D:91 | *X-109937582 - AX-110605376* | 549.19-557.58 | 12.29 | 18.88 | -2.12 |
|  |  | 2019GHS | 7D:91 | *X-109937582 - AX-110605376* | 549.19-557.58 | 26.15 | 38.25 | -2.52 |
|  |  | 2017CSS | 7D:91 | *X-109937582 - AX-110605376* | 549.19-557.58 | 13.51 | 19.27 | -1.46 |
|  |  | 2018CSS | 7D:91 | *X-109937582 - AX-110605376* | 549.19-557.58 | 19.48 | 28.66 | -1.86 |
| SFW | *QSfw.saas-1DS* | 2017GHS | 1DS:33 | *AX-94812958 - AX-109908110* | 8.97-11.57 | 3.70 | 7.51 | -8.98 |
|  |  | 2018GHS | 1DS:34 | *AX-94812958 - AX-109908110* | 8.97-11.57 | 6.40 | 11.31 | -11.17 |
|  |  | 2019GHS | 1DS:36 | *AX-94812958 - AX-110910133* | 8.97-21.51 | 7.23 | 12.63 | -10.11 |
|  |  | 2017CSS | 1DS:42 | *AX-94812958 - AX-110910133* | 8.97-21.51 | 2.96 | 8.03 | -8.26 |
|  |  | 2018CSS | 1DS:36 | *AX-94812958 - AX-110910133* | 8.97-21.51 | 4.27 | 8.71 | -10.30 |
|  | *QSfw.saas-7D* | 2017GHS | 7D:91 | *AX-109937582 - AX-110605376* | 549.19-557.58 | 4.83 | 9.13 | -10.13 |
|  |  | 2018GHS | 7D:91 | *AX-109937582 - AX-110605376* | 549.19-557.58 | 9.07 | 16.25 | -13.70 |

Marker interval	Physical interval

LOD	PVE

ADDa

|  | 2019GHS | 7D: 91 | *AX-109937582 - AX-110605376* | 549.19-557.58 | 15.97 | 26.61 | -15.88 |
| --- | --- | --- | --- | --- | --- | --- | --- |
|  | 2017CSS | 7D: 91 | *AX-109937582 - AX-110605376* | 549.19-557.58 | 6.03 | 10.17 | -10.93 |
|  | 2018CSS | 7D:91 | *AX-109937582 - AX-110605376* | 549.19-557.58 | 7.66 | 14.08 | -14.22 |
| SDW *QSdw.saas-1DS* | 2017GHS | 1DS:40 | *AX-94812958 - AX-110910133* | 8.97-21.51 | 4.35 | 10.59 | -1.39 |
|  | 2018GHS | 1DS:34 | *AX-94812958 - AX-109908110* | 8.97-11.57 | 6.93 | 12.28 | -1.53 |
|  | 2019GHS | 1DS:36 | *AX-94812958 - AX-110910133* | 8.97-21.51 | 6.98 | 12.84 | -1.40 |
|  | 2017CSS | 1DS:40 | *AX-94812958 - AX-110910133* | 8.97-21.51 | 2.73 | 7.24 | -1.02 |
|  | 2018CSS | 1DS:35 | *AX-94812958 - AX-110910133* | 8.97-21.51 | 3.93 | 7.79 | -1.28 |
| *QSdw.saas-7D* | 2017GHS | 7D:89 | *AX-109917900 - AX-110605376* | 324.36-557.58 | 4.40 | 6.53 | -1.22 |
|  | 2018GHS | 7D:91 | *AX-109937582 - AX-110605376* | 549.19-557.58 | 8.24 | 14.81 | -1.71 |
|  | 2019GHS | 7D:91 | *AX-109937582 - AX-110605376* | 549.19-557.58 | 13.10 | 22.20 | -2.00 |
|  | 2017CSS | 7D:91 | *AX-109937582 - AX-110605376* | 549.19-557.58 | 5.77 | 10.09 | -1.38 |
|  | 2018CSS | 7D:91 | *AX-109937582 - AX-110605376* | 549.19-557.58 | 6.88 | 13.24 | -1.76 |

262 aAdditive effect. Positive and negative, Syn79 and Syn80 allele produced larger value, respectively.
263 SDW is positively related to SFW at seedling. In this study, we detected two QTL for
264 both SFW and SDW on chromosomes 1DS and 7D (Table 6; Fig 3). And the QTL intervals
265 for SFW and SDW were in accordance with the QTL intervals for both PH and TN. Since
266 higher plant height and more tiller number per plant resulted in larger SFW and SDW, it
267 suggested that they may be the same QTLs. The average PVEs for *QSfw.saas-1DS* and
268 *QSdw.saas-1DS* were 9.64 % and 10.15%, respectively. And the QTL allele from Syn80
269 increased the SFW and SDW (Table 6). In the interval of *AX-109937582- AX-110605376* on
270 chromosome 7D, QTL for both SFW and SDW were identified under all five environmental
271 conditions, and the average PVE of *QSfw.saas-7D* and *QSdw.saas-7D* were 15.25 % and
272 13.37 %, respectively (Table 6). And the QTL alleles increasing SFW and SDW were from
273 the parent Syn80 (Table 6).
274 In this study, about two genomic regions were identified to be associated with early
275 biomass. They were in the interval of *AX-94812958 - AX-110910133* on chromosome 1DS
276 and the interval of *AX-109917900 - AX-110605376* on chromosome 7D, respectively. The
277 two genomic regions from the parent Syn80 could significantly enhance the early biomass
278 with pleiotropic effects of increasing PH, TN, SFW and SDW.

# 279 Discussion

280 Greater early biomass is visual and important to breed new variety and innovative utilize
281 crop germplasm, especially under adverse environmental conditions. Therefore, it is an
282 important selective trait under drought stress [29-31], especially in Sichuan, where drought or
283 seasonal drought occurred frequently in the last 70 years [32]. In this study, early biomass of
284 parents and RIL population showed significant phenotypic difference on PH, TN, SFW and
285 SDW under the five different environmental conditions from 2016 to 2019. Phenotypic and
286 QTL analysis demonstrated that early biomass related traits PH, TN, SFW and SDW were
287 controlled by polygenes. Wheat growth habit types (spring or winter), the wheat growth
288 progress and early biomass were affected by the combination of photoperiod and
289 vernalization genes [33-36]. Photoperiod and vernalization genes on D genome were located
290 on 2D and 5D [33,34]. Considering that the phenology and growing periods of two parents
291 and RILs were consistent, it can be inferred that early biomass in this RILs was controlled by
292 genes, which could not be related to photoperiod or vernalization genes, for no QTL detected
293 on the chromosome 2D or 5D.
294 In the present study, two synthetic wheats Syn79 and Syn80 were generated from two
295 different *Ae. tauschii* accessions crossing with the same tetraploid wheat, and the significant
296 difference of early biomass between them was caused by their different D genome donors. *Ae.*
297 *tauschii*, the D genome donor of common wheat, exhibited genetic diversity on early growth
298 and might be a valuable species for improvement of early vigor in wheat [37]. The common
299 wheat D genome progenitor *Ae. tauschii* showed a rapid leaf expansion rate at the seedling
300 stage [21,37], which is beneficial for reducing evaporative losses from the soil [21]. Genetic
301 dissection for early vigor related traits has been reported in several germplasm under different
302 growing conditions, and QTL for early vigor related traits were distributed through almost
303 the whole genome of wheat [21,37-41]. ter Steege et al identified 87 QTL for early growth
304 related with 33 traits, 3.1 QTL per trait, explaining 32% of the phenotypic variance by using
305 population of *Ae. tauschii* RILs at seedling stage, but there was no significant QTLs for plant
306 and shoot mass could be detected in his study, considering that the effects of QTL for the
307 underlying growth traits counterbalanced each other [37]. However, in our study, the two
308 chromosome fragments for SFW and SDW were detected, which simultaneously regulated
309 PH and TN. And the favorable alleles detected were from *Ae. tauschii* and they could express
310 stably in a hexaploid genetic background. Few QTLs for biomass have been identified in the
311 diploid	populations	of	*Ae. tauschii*, but	in	a	hexaploidy genetic
312 background. The AABB genome of tetraploid wheat may play a very important role in
313 synthetic wheat of tetraploid wheat and *Ae. tauschii*. The effects of genome combination
314 between AABB and DD for genes expression need to be analyzed further. And it
315 substantiates the conclusion that using SHW is a more effective method to transfer favor genes from *Ae.*
316 *tauschii* to common wheat [6,7,9,42].
317 In addition, the two chromosome fragments for PH, TN, SFW and SDW were detected
318 stably on 1DS and 7D, which were located on the genomic interval of 8.98 - 21.51 Mb and
319 324.36 - 557.58 Mb, respectively. *Lr42*, *Rmg6*, *Sr33*, *SrTA1662*, *LR10*, *Xa5, Chalk5*, *MHZ5*,
320 *B10*, *Rc*, *BC10*, *EBR1* and *EBR1* were located in the interval of *AX-94812958*
321 *-AX-110910133* on 1DS of *Ae. tauschii*, and 16 QTL/genes (*Pid2, IPA1, Xa13, Hd18, GW8,*
322 *Xa27-Xa27-IRBB27, qUVR-10, Yr33, Dn2, Ehd3, Nud, OsABCG15, MOC1, Lks2, TaD27* and
323 QTls for antixenosis were in the interval of *AX-109917900 -AX-110605376* on 7D 0. And
324 among these reported genes, no one except for *TaD27* associated to tiller number in
325 hexaploidy has been found to be related to early vigor sa far, indicating that QTLs for early
326 biomass on 1DS in this population were new genes located in this study.
327 Segregation distortion is a common phenomenon among many plants 0. In the present
328 study, 54 of 153 SNP loci showed segregation distortion in RILs, and 50 makers were
329 skewed to Syn80 and 4 biased to Syn79. Segregation distortion loci accounted for 35.29 % of
330 the total polymorphic loci, and 92.59 % loci preferred female parent Syn80, only 7.41 %
331 came from male parent Syn79. At the same time, we found that Syn80 had stronger seedling
332 vigor than one of Syn79. Therefore, the early vigor providing high survival ratio of the RILs
333 containing Syn80 loci was higher than that of the RILs containing the Syn79 loci. The
334 proportion of segregation distortion was high in RILs. Xu et al found the similar phenomenon,
335 which was the purer population and higher separation ratio 0. In the present study, three
336 genomic regions were detected to be Syn80-biased on chromosome 1D, 2D and 7D (Table 5),
337 which were involved with the QTL intervals for early biomass. And the center of segregation
338 bias on chromosome 1DS was located in the interval of *AX-110090502-AX-109911195* with
339 genetic location from 8.26 cM to 12.44 cM, as 96.4 % of the progeny shared the same
340 genotype with the parent Syn80 at the SNP site of *AX-110090502* and 97.9 % for
341 *AX-109911195*. On chromosome 2D, the segregation bias region was framed from
342 *AX-108911375* to *AX-110935958* across about 20 cM. And on 7D, the center of segregation
343 bias was located in the interval of *AX-110271371* to *AX-94807766*. The center of segregation
344 bias on 1DS was about 25 cM away from the detected QTL peaks for early growth-related
345 traits, and the center of segregation bias on 7D was just located in the interval of QTL peaks
346 detected on this chromosome. Up to now, many factors may cause segregation distortion，
347 which may be genetic factor such as reproductive isolation, or incoordination between
348 cytoplasm and nucleus, or hybrid necrosis and so on 0, and it may be due to natural or
349 artificial selection 0. In most cases, segregation is controlled by reproductive isolation factors
350 such as gametophyte genes on the nucleus or sterility genes [48-51]. As several types of
351 hybrid abnormalities including hybrid necrosis were reported in the process of synthetic
352 wheat production 0. Usually, this abnormal growth phenotypes are often classified into
353 hybrid necrosis (Types II and III), hybrid chlorosis and severe growth abortion 0]. And two
354 genes derived from *Ae.tauschii* related to type II and III necrosis symptoms have been
355 mapped 0. The gene *Nec1* of type III necrosis was on chromosome 7DS 0, while the gene
356 *Nec2* of type II necrosis was on chromosome 2DL 0. The location of *Nec2* and *Nec1* were
357 closed to the segregation bias region on chromosome 2D and the segregation bias center on
358 chromosome 7D. And the reason caused the segregation bias to Syn80 in these loci was that
359 Syn79 may carried the *Nec2* and *Nec1* alleles for hybrid necrosis. Thus, the situation of
360 segregation bias would spread from the location of *Nec2* or *Nec1* across the QTL regions in
361 this population. Segregation distortion regions may be related to certain genes, the gene
362 location of the target trait can be preliminarily determined according to segregation distortion
363 region of the genetic map and the phenotypic data. However, no strong evidence showed the
364 early biomass QTL was caused by segregation bias to Syn80.
365 In the present study, 3480 SNP markers were used on the D genome, and only 153
366 polymorphic markers were detected between parents, a percent polymorphism of
367 4.40%. Comparing to the genetic diversity of *Ae. taushcii* and wheat cultivars reported by
368 previous authors [56-58], one of Syn79 and Syn80 was low on D gennome. It has been
369 widely accepted that *Ae. tauschii* ssp. *strangulata* is the D genome donor of hexaploid wheat
370 [56,59-63]. *Ae. tauschii* was classified into two groups, lineage 1 and lineage 2 [56,64]. Lineage 1
371 is broadly related to *Ae. tauschii* ssp*. tauschii* and lineage 2 is broadly related to *Ae. tauschii*
372 ssp*. strangulata*. The Infinium SNP array for D genome was developed mainly according to
373 the SNP polymorphism between *Ae. tauschii* ssp. *tauschii* and *Ae. tauschii* ssp. *strangulata*.
374 Therefore, the D genome donors (AT333 and AT428) of synthetic hexaploid wheat Syn79
375 and Syn80 may belong to the same group (Lineage 1 or Lineage 2), and their genetic
376 relationship is very close. Although the polymorphic loci of D genome between Syn79 and
377 Syn80 was low, two genome regions on 1DS and 7D for four early biomass related traits
378 were still detected under five different environments. It provided a basis for further fine
379 mapping and candidate gene analysis of a few QTLs of early biomass related traits. On the
380 other hand, each of the synthetic wheat Syn79 and Syn80 having newly genes from tetraploid
381 wheat and *Ae. tauschii* is a potential resource to broaden the genetic diversity for wheat
382 breeding programs.
383 383

# 384 Conclusion

385 By using a set of recombinant inbred lines derived from two synthetic hexaploid wheats
386 (Syn79 and Syn80) re-synthesized from the same tetraploid wheat DOY1 and two different
387 *Ae. tauschii* accessions (AT333 and AT428), two genomic regions on 1DS and 7D were
388 detected to be associated with early biomass, with pleiotropic effects on PH, TN, SFW and
389 SDW. The QTL alleles from Syn80 enhanced the early biomass with increasing PH, TN,
390 SFW and SDW, and originated from the *Ae. tauschii* AT428, which could express stably in a
391 hexaploid background. The framed SNP markers could be used for wheat improvement.
392 392

# 393 Acknowledgments

394 We thank the International Maize and Wheat Improvement Center for providing
395 synthetic hexaploid wheat parents (Syn79 and Syn80). National Natural Science Foundation
396 of China and Department of Science and Technology of Sichuan Province supported this
397 work. And we thank our colleagues and staffs at Guang-Han Station and Cang-shan
398 398
399 399

Station of Sichuan Academy of Agricultural Sciences for helps

in this study.

# 400 REFERENCES

401 1. Kihara H. Discovery of the DD-analyser, one of the ancestors of *Triticum vulgare.* Agric

402	Hortic. 1944;19:889-890.

403	2. McFadden ES, Sears ER. The origin of *Triticum spelta* and its free-threshing hexaploid

404	relatives. J Hered. 1946;37:107-116.

405 3. Bultynck L, ter Steege MW, Schortemeyer M, Poot P, Lambers H. From individual leaf
406 elongation to whole shoot leaf area expansion;a comparison of three *Aegilops* and two
407 *Triticum* species. Ann Bot (Lond). 2004;94:99–108.
408 4. Luo MC, Yang ZL, You FM, Kawahara T, Waines JG, Dvorak J. The structure of wild and
409 domesticated emmer wheat populations, gene flow between them, and the site of emmer
410 domestication. Theor Appl Genet. 2007;6:947–959.
411 5. Dvorak J, Luo MC, Yang ZL, Zhang HB. The structure of the *Aegilops tauschii* genepool
412 and the evolution of hexaploid wheat. Theor Appl Genet. 1998;4:657–670.
413 6. Mujeeb-Kazi A, Rosas V, Roldan S. Conservation of the genetic variation of *Triticum*
414 *tauschii (Coss.) Schmalh*. (*Aegilops squarrosa* auct. non. L) in synthetic hexaploid wheats
415 (*T. turgidum* L. s. lat × *T. tauschii*; 2n = 6x = 42, AABBDD) and its potential utilization
416 for wheat improvement. Genet Resour Crop. Evol. 1996;43:129–134.
417 7. Das MK, Bai GH, Mujeeb-Kazi A, Rajaram S. Genetic diversity among synthetic
418 hexaploid wheat accessions (*Triticum aestivum*) with resistance to several fungal diseases.

419	Genet. Resour Crop Evol. 2016;8:1285-1296.

420 8. Rana RM, Bilal M, Rehman SU, Iqbal F, Shah MKN. Synthetic wheat: a new hope for the
421 hungry world. Asian J Agric Bio. 2013;1:91-94.
422 9. Yang WY, Liu DC, Li J, Zhang LQ, Wei HT, Hu XR, et al. Synthetic hexaploid wheat and
423 its utilization for wheat genetic improvement in China. J Gene Genomics.2009;9:539-546.
424 10. Ren Q, Liu HJ, Zhang ZY, Feng J, Xu SC, Pu ZJ, et al. Characterization and molecular
425 mapping of a stripe rust resistance gene in synthetic wheat C1110. J Integ. Agr.

426	2012;4:521-527.

427	11. Mulki MA, Jighly A, Ye G, Emebiri LC, Moody D, Ansari O, et al. Association mapping

428	for	soilborne	pathogen	resistance	in	synthetic	hexaploid	wheat.	Mol	Breed.

429	2013;2:299-311.

430	12. Bouhssini ME, Ogbonnaya FC, Chen M, Lhaloui S, Rihawi F, Dabbous A. Sources of

431	resistance in primary synthetic hexaploid wheat (*Triticum aestivum* L.) to insect pests:

432	*Hessian fly*, Russian wheat aphid and Sunn pest in the fertile crescent. Genet Resour Crop

433	Evol. 2013;2:621-627.

434 13. Van Ginkel M, Ogbonnaya F, Imtiaz M, Ramage C, Borgognone MG, Dreccer F, et al.
435 Molecular breeding for salt tolerance, pre-harvest sprouting resistance and disease
436 resistance using synthetic hexaploid wheats, genetic transformation, and associated
437 molecular markers. Wheat Production in Stressed Environments. 2007;12:383-385.
438 14. Li AL, Liu DC, Yang WY, Kishii M, Mao L. Synthetic hexaploid wheat: yesterday, today,
439 and tomorrow. Engineering. 2018;4:552-558.
440 15. Yang WY, Yen C, Yang JL, Zheng YL, Liu DC. Evaluation of shape *Aegilops tauschii*
441 Coss for resistance to physiological strains *CYR30* and *CYR31* of wheat stripe rust in
442 China. Genet Resour Crop Evol. 1998;45:395–398.
443 16. Li CS, Wu XL, Tang YL, Yang WY, Wu YQ, Wu C, et al. Quality of major wheat
444 cultivars grown in Sichuan province in recent decade. Acta Agron Sin. 2016;6:803-812
445 (in Chinese with English abstract).
446 17. Ni ZF, Zhang YR, Liang RQ, Liu GT, Sun QX. Genetic diversity of D-genome revealed
447 by SSR markers in synthesized hexaploid wheat Introduced from CIMMYT. Acta
448 Genetica Sinica. 2002;6:542-548 (in Chinese with English abstract).
449 18. Tang YL, Yang WY, Wei HT, Li CS, Li J. Opportunities for breaking the barriers of
450 wheat yield using synthetic hexaploid wheats. Acta Scientiarum Naturalium Universitatis
451 Sunyatseni. 2010;3:3465-3476 (in Chinese with English abstract).
452 19. Li J, Wan HS, Yang WY. Synthetic hexaploid wheat enhances variation and adaptive
453 evolution of bread wheat in breeding processes. J Syst Evol

454	2014;52:735–742.

455 20. Wan HS, Yang YM, Li J, Zhang ZF, Yang WY. Mapping of a major QTL for hairy leaf
456 sheath introgressed from *Aegilops tauschii* and its association with enhanced grain yield
457 in bread wheat. Euphytica. 2015;205:275-285.
458 21. Li J, Wan HS, Wei HT, Wang Q, Zhou YH, Yang WY. QTL mapping for early vigor
459 related traits in an elite wheat-breeding parent Chuanmai 42 derived from synthetic
460 hexaploid wheat. Pak J Agri Sci. 2017;55(1):33-45.
461 22. Ryan PR, Liao M, Delhaize E, Rebetzke GJ, Weligama C, Spielmeyer W et al. Early
462 vigour improves phosphate uptake in wheat. J Exp Bot. 2015;66:7089–7100.
463 23. Guo Y, Kong FM, Xu YF, Zhao Y, Liang X, Wang YY, et al. QTL mapping for seedling
464 traits in wheat grown under varying concentrations of N, P and K nutrients, Theor Appl

465	Genet. 2012;124: 851-865.

466 24. Yang YM, Yang WY, Wan HS, Zhang J, Li J, Lei JR, et al. Difference in growth and
467 nutrient accumulation of synthetic wheat with different D genome under nitrogen,
468 phosphorus	and	potassium	stresses.	J	of	Plant	Nutrition	and	Fertilizer.
469 2015;5:1123-1131(in Chinese with English abstract).
470 25. Meng L, Li HH, Zhang LY, Wang JK. QTL IciMapping: integrated software for genetic
471 linkage map construction and quantitative trait locus mapping in biparental populations.

472	Crop J. 2015;3:269-283.

473 26. Li HH, Ye GY, Wang JK. A modified algorithm for the improvement of composite
474 interval mapping. Genetics. 2007;175:361-374.
475 27. Luo MC, Gu YQ, Puiu D, Wang H, Twardziok SO, Deal KR, et al. Genome sequence of
476 the progenitor of the wheat D genome *Aegilops tauschii.* Nature. 2017;551:498-502.
477 28. Ayalew H, Liu H, Liu CJ, Yan GJ. Identification of early vigor QTLs and QTL by
478 environment interactions in wheat (*Triticum eastivum* L.). Plant Mol Biol Rep.

479	2018;36:399-405.

480	29. Passioura JB. Drought and drought tolerance. Plant Growth Regul. 1996;20:79-83.

481	30. Rebetzke GJ, Bruce SE, Kirkegaard JA. Longer coleoptiles improve emergence through

482	crop residues to increase seedling number and biomass in wheat (*Triticum aestivum* L.).

483	Plant Soil. 2005;272:87-100.

484 31. Botwright TL, Condon AG, Rebetzke GJ, Richards RA. Field evaluation of early vigour
485 for genetic improvement of grain yield in wheat. Aust J Agric Res. 2002;53:1137-1145.
486 32. Deng SH, Luo XB. Features, prevention and remedy of droughts in Sichuan Since 1949.
487 Journal of Sichuan Normal University (Social Sciences Edition). 2005;3:125-132(in
488 Chinese with English abstract).
489 33. Yoshida T, Nishida H, Zhu J, Nitcher R, Distelfeld A, Akashi Y, et al. *Vrn-D4* is a
490 vernalization gene located on the centromeric region of chromosome 5D in hexaploid
491 wheat. Theor Appl Genet. 2010;120:543-552.
492 34. Beales J, Turner A, Griffiths S, Snape JW, Laurie DA. A Pseudo-Response Regulator is
493 misexpressed in the photoperiod insensitive *Ppd-D1a* mutant of wheat (*Triticum aestivum*

494	L.). Theor Appl Genet. 2007;115:721-733.

495	35. Gororo NN, Flood RG, Eastwood RF, Eagles HA. Photoperiod and Vernalization

496	Responses in *Triticum turgidum* x *T. tauschii* Synthetic Hexaploid Wheats. Ann

497	Bot. 2001;88:947–952.

498	36. Dubcovsky J, Loukoianov A, Fu DL, Valarik M, Sanchez A and Yan LL. Effect of

499	photoperiod on the regulation of wheat vernalization genes *VRN1* and *VRN2*. Plant

500	Mol Biol. 2006;60:469–480.

501 37. ter Steege MW, den Ouden FM, Lambers H, Stam P, Peeters AJM. Genetic and
502 physiological architecture of early vigor in *Aegilops tauschii*, the D-genome donor of
503 hexaploid wheat. a quantitative trait loci analysis. Plant Physiol. 2005;139:1078–1094.
504 38. Li J, Wan HS, Wei HT, Wang Q, Zhou YH, Yang WY. QTL mapping for early vigor
505 related traits in an elite wheat-breeding parent Chuanmai 42 derived from synthetic
506 hexaploid wheat. Pak J Agri Sci. 2017;55:33-45.
507 39. Su JY, Zheng Q, Li HW, Li B, Jing RL, Tong YP, Li ZS. Detection of QTLs for
508 phosphorus use efficiency in relation to agronomic performance of wheat grown under
509 phosphorus sufficient and limited conditions. Plant Sci. 2009;176:824–836.
510 40. Genc Y, Oldach K, Verbyla AP, Lott G, Hassan M, Tester M, et al. Sodium exclusion
511 QTL associated with improved seedling growth in bread wheat under salinity stress.

512	Theor Appl Genet. 2010;121:877-894.

513	41. Guo Y, Kong FM, Xu YF, Zhao Y, Liang X, Wang YY, et al. QTL mapping for seedling

514	traits in wheat grown under varying concentrations of N, P and K nutrients. Theor Appl

515	Genet. 2012;124:851-865.

516 42. Li AL, Liu DC, Wu J, Zhao XB, Hao M, Geng SF, et al. mRNA and small RNA
517 transcriptomes reveal insights into dynamic homoeolog regulation of allopolyploid
518 heterosis in nascent hexaploid wheat. Plant Cell. 2014;26:1878–1900.
519 43. Zhao GY, Zou C, Li K, Wang K, Li TB, Gao LF, et al. The *Aegilops tauschii* genome
520 reveals multiple impacts of transposons. Nature Plants. 2017;3:946-955.
521 44. Takumi S, Motomura Y, Iehisa JCM, Kobayashi F. Segregation distortion caused by
522 weak hybrid necrosis in recombinant inbred lines of common wheat. Genetica.

523	2013;141:463.

524	45. Xu Y, Zhu L, Xiao J, Huang N, McCouch SR. Chromosomal regions associated with

525	segregation distortion of molecular markers in F2	back-cross double haploid and

526	recombinant	inbred	populations	in	rice	*(Oryza	sativ*a	L	).	Mol	Gen	Genet.

527	1997;253:535-545.

528 46. Goloenko IM, Davydenko OG, Shimkevich AM. Segregation distortion of marker nuclear
529 genes in alloplasmic and isoplasmic lines of barley. Russ J Genet. 2002;38:791-795.
530 47. Wang YJ, Wu XL, Yu DY, Zhang YM, Chen SY, Gai JY. Method of evaluation and
531 ajustment of recombinant inbred line population and its application to the soybean RIL
532 population NJRIKY. Acta Agronomica Sinica. 2004;30:413-418 (in Chinese with English
533 abstract).
534 48. Zhao B, Deng QM, Zhang QJ, Li JQ, Ye SP, Liang YS, et al.. Analysis of segregation
535 distortion of molecular markers in F2 population of rice. Acta Genetica Sinica.

536	2006;33:449-457.

537 49. He P, Li JZ, Zheng XW, Shen LS, Lu MF, Chen Y, et al. Comparison of molecular
538 linkage maps and agromomic trait loci between DH and RIL population derived from the
539 same rice cross. Crop Sci. 2001;41:1240-1246.
540 50. Harushima Y, Nakagahra M, Yano M, Sasaki T, Kurata N. Diverse variation of
541 reproductive barriers in three intraspecific rice crosses. Genetics. 2002;160:313-322.
542 51. Harushima Y, Kurata N, Yano M, Nagamura Y, Sasaki T, Minobe Y,et al. Detection of
543 segregation distortions in an indica-japonica rice cross using a high-resolution molecular

544	map. Theor Appl Genet. 1996;92:145-150.

545 52. Nishikawa K. Hybrid lethality in crosses between Emmer wheats and *Aegilops squarrosa,*
546 II. Synthesized 6x wheatis employed as test varieties. Jpn J Genet. 1962;37:227–236.
547 53. Matsuoka Y, Takumi S, Kawahara T. Natural variation for fertile triploid F1 hybrid
548 formation in allohexaploid wheat speciation. Theor Appl Genet. 2007;115:509–518.
549 54. Mizuno N, Hosogi N, Park P, Takumi S. Hypersensitive response-like reaction is
550 associated with hybrid necrosis in interspecific crosses between tetraploid wheat and
551 *Aegilops	tauschii*	Coss.	PloS	One.	2010;5(6):e11326.
552 https://doi.org/10.137/journal.pone.0011326.
553 55. Ryusuke M, Julio CMI and Shigeo T. Application of real-time PCR-based SNP detection
554 for mapping of *Net2*, a causal D-genome gene for hybrid necrosis in interspecific crosses
555 between tetraploid wheat and *Aegilops tauschii*. Genes Genet Syst. 2012;87:137–143.
556 56. Wang JR, Luo MC, Chen ZX, You FM, Wei YM, Zheng YL, et al. *Aegilops tauschii*
557 single nucleotide polymorphisms shed light on the origins of wheat D–genome genetic
558 diversity and pinpoint the geographic origin of hexaploid wheat. New Phytol.

559	2013;198:925–937.

560 57. Cao TJ, Xie TZ, Wu QH, Chen YX, Wang ZZ, Zhao H, et al. Genetic diversity of
561 registered wheat varieties in Henan province based on pedigree and single-nucleotide
562 polymorphism. Acta Agronomica Sinica. 2015;41:197-206 (in Chinese with English
563 abstract).
564 58. Liu YK, Zhu ZW, Chen L, Zou J, Tong HW, Zhu G, et al. Revealing the genetic diversity
565 of wheat varieties (lines) in China based on SNP markers. Acta Agronomica Sinica.
566 2019[;http://kns.cnki.net/kcms/detail/11.1809.S.20190910.1511.012.html (in Chinese with](http://kns.cnki.net/kcms/detail/11.1809.S.20190910.1511.012.html)
567 [English abstract).](http://kns.cnki.net/kcms/detail/11.1809.S.20190910.1511.012.html)
568 59. Nishikawa K. Alpha-amylase isozymes and phylogeny of hexaploid wheat. In: Sears ER,
569 Sears LMS, eds. 4th International Wheat Genetics Symposium. Columbia, MO, USA:
570 Missouri Agr. Exp. Sta. 1973; 851–855.
571 60. Nakai Y. Isozyme variation in *Aegilops* and *Triticum*, IV. The origin of the common
572 wheats revealed from the study on esterase isozymes in synthesized hexaploid wheats.
573 Japan Journal of Genetics. 1979;54:175–189.
574 61. Lubbers EL, Gill KS, Cox TS, Gill BS. Variation of molecular markers among
575 geographically diverse accessions of *Triticum tauschii*. Genome. 1991;34:354–361.
576 62. Dvorak J, Luo MC, Yang ZL. Restriction fragment length polymorphism and divergence
577 in the genomic regions of high and low recombination in self-fertilizing and
578 cross-fertilizing *Aegilops* species. Genetics. 1998;148:423–434.
579 63. Dvorak J, Deal KR, Luo MC, You FM, von Borstel K, Dehghani H. The origin of spelt
580 and free-threshing hexaploid wheat. J Hered. 2012;103: 426–441.
581 64. Mizuno N, Yamasaki M, Matsuoka Y, Kawahara T, Takumi S. Population structure of
582 wild wheat D-genome progenitor *Aegilops tauschii* Coss.: implications for intraspecific
583 lineage	diversification	and	evolution	of	common	wheat.	Mol	Ecol.

584	2010;19:999–1013.

Figure	[Click here to download Figure Fig 1..tiff](https://www.editorialmanager.com/pone/download.aspx?id=26045930&guid=162203e6-dba4-412e-9e02-cda1efddd36a&scheme=1)

[.:!](https://www.editorialmanager.com/pone/download.aspx?id=26045930&guid=162203e6-dba4-412e-9e02-cda1efddd36a&scheme=1)

RJL121	RILl4l	Syn,80	RJL,51	RI Ll.48 Syn79	R IL l.18	RILJO

i

i

Figure	[Click here to download Figure Fig 2..tif](https://www.editorialmanager.com/pone/download.aspx?id=26045931&guid=a4b72d2a-72a9-4afe-b680-8042aefa3b57&scheme=1)

Figure	[Click here to download Figure Fig 3..tif](https://www.editorialmanager.com/pone/download.aspx?id=26045932&guid=42b45108-a95f-40c0-918c-4e5cc7e7790e&scheme=1)
