# Supplementary material for: Mapping QTLs for enhancing early biomass derived from Aegilops tauschii in synthetic hexaploid wheat
Source: PLoS One. 2020 Jun 25;15(6):e0234882. doi: 10.1371/journal.pone.0234882 (PMC7316292; doi:10.1371/journal.pone.0234882)
Supplement: Attachment — Submitted filename: Response to Reviewers.doc [file pone.0234882.s006.doc]

Point-to-point reply

Dear Dr Zhang and Reviewers,

Thank you very much for giving us an opportunity to revise our manuscript, we appreciate editor and reviewers very much for their positive and constructive comments and suggestions on our manuscript. We have accepted all the modifications made by the two reviewers, and answered the comments carefully. We have tried our best to revise our manuscript according to the reviewers’ comments and PLOS ONE’s style requirements. In addition, this manuscript was edited for proper English language, grammar, punctuation, spelling, and overall style by one or more of the highly qualified native English speaking editors at NativeEE. We have made revision which marked in red in the paper.

We would like to express our great appreciation to you and reviewers for comments on our paper.

Looking forward to hearing from you.

Thank you and best regards.

Yours sincerely,

Wuyun Yang

List of responses:

1) Please ensure that your manuscript meets PLOS ONE's style requirements, including those for file naming.

Response:

Thanks for your reminder. We carefully have read PLOS ONE’s style requirements again, and carefully modified to meet its style requirements.

2) We suggest you thoroughly copyedit your manuscript for language usage, spelling, and grammar.

Response:

Thanks for your suggestion. Native English speaking editors at Native English Editing checked and corrected for English language, spelling, grammar.

Upon resubmission, please provide the following:

- The name of the colleague or the details of the professional service that edited your manuscript
- A copy of your manuscript showing your changes by either highlighting them or using track changes (uploaded as a *supporting information* file)
- A clean copy of the edited manuscript (uploaded as the new *manuscript* file)

Response:

OK. We will resubmit the manuscript according to your requirements.

Our manuscript for language usage, spelling, and grammar were checked and corrected by native English speaking editors at Native English Editing, which provided a statement of editing. We uploaded as separate file and labeled ‘Statement of Editing’.

We showed our changes in our manuscript by in red font. The file was uploaded separately and labeled ‘Revised Manuscript with Track Changes’.

We uploaded a clean manuscript separately, this file was labeled ‘Manuscript’.

3) PLOS requires an ORCID iD for the corresponding author in Editorial Manager on papers submitted after December 6th, 2016. Please ensure that you have an ORCID iD and that it is validated in Editorial Manager. To do this, go to ‘Update my Information’ (in the upper left-hand corner of the main menu), and click on the Fetch/Validate link next to the ORCID field. This will take you to the ORCID site and allow you to create a new iD or authenticate a pre-existing iD in Editorial Manager. Please see the following video for instructions on linking an ORCID iD to your Editorial Manager account: <https://www.youtube.com/watch?v=_xcclfuvtxQ>

[Note: HTML markup is below. Please do not edit.]

Response:

OK. The corresponding author Wuyun Yang has an ORCID iD, and it is validated in Editorial Manager.

Reviewers' comments:
Reviewer's Responses to Questions
**Comments to the Author**
1. Is the manuscript technically sound, and do the data support the conclusions?
The manuscript must describe a technically sound piece of scientific research with data that supports the conclusions. Experiments must have been conducted rigorously, with appropriate controls, replication, and sample sizes. The conclusions must be drawn appropriately based on the data presented.
Reviewer #1: Yes
Reviewer #2: Yes

Response:

Thanks for your recognition.

2. Has the statistical analysis been performed appropriately and rigorously?
Reviewer #1: Yes
Reviewer #2: Yes

Response:

Thanks for your recognition.

3.Have the authors made all data underlying the findings in their manuscript fully available?
The [PLOS Data policy](http://www.plosone.org/static/policies.action#sharing) requires authors to make all data underlying the findings described in their manuscript fully available without restriction, with rare exception (please refer to the Data Availability Statement in the manuscript PDF file). The data should be provided as part of the manuscript or its supporting information, or deposited to a public repository. For example, in addition to summary statistics, the data points behind means, medians and variance measures should be available. If there are restrictions on publicly sharing data—e.g. participant privacy or use of data from a third party—those must be specified.
Reviewer #1: Yes
Reviewer #2: Yes

Response:

Thanks for your recognition.

4. Is the manuscript presented in an intelligible fashion and written in standard English?
PLOS ONE does not copyedit accepted manuscripts, so the language in submitted articles must be clear, correct, and unambiguous. Any typographical or grammatical errors should be corrected at revision, so please note any specific errors here.
Reviewer #1: Yes
Reviewer #2: No

Response:

We have tried our best to modify the manuscript. At the same time, this manuscript was edited for proper English language, grammar, punctuation, spelling, and overall style by one or more of the highly qualified native English speaking editors at NativeEE. We hope that the revised manuscript can meet PLOS ONE’s requirements.

5. Review Comments to the Author
Please use the space provided to explain your answers to the questions above. You may also include additional comments for the author, including concerns about dual publication, research ethics, or publication ethics. (Please upload your review as an attachment if it exceeds 20,000 characters)

Reviewer #1: This manuscript described analysis of QTLs related to early biomass using a RIL population derived from two synthetic hexaploid wheat with same AABB-genome background. Four stable QTL for plant height (PH), tiller number (TN), shoot fresh weight (SFW) and shoot dry weight (SDW) were detected on chromosomes 1D and 7D. The manuscript presented some interesting results using synthetic hexaploid wheat accessions distinguished by only D genome. I felt this manuscript is valuable because that diversity of wheat D genome is very low. The introduction of novel D-derived genes through synthetic hexaploid is a efficient way to expand genetic background of bread wheat. The identification of novel QTLs for early biomass from *Aegilops tauschii* are valuable for wheat improvement. It will be of interest for the readers of Plos One. Thus, this manuscript is acceptable, but there are some problem needs to be addressed.
1)There are some witting errors needs thorough correction, some were marked on the pdf. And the language also need improve.

Response:

Thank you for your careful revision. We have accepted your correction. We have tried our best to modify the manuscript. At the same time, this manuscript was edited for proper English language, grammar, punctuation, spelling, and overall style by one or more of the highly qualified native English speaking editors at NativeEE. We hope that the revised manuscript can meet PLOS ONE’s requirements.

2)Some statistical information need to reconfirm and replenish.
P10-L181-183: The authors claim that the H^2^ =0.4327 for PH is significant lower than the results of previous reported. I suggested that the authors perform an ANOVA again to confirm whether the error variance is too large or the calculation is wrong.

Response:

In this study, the PH was investigated at the jointing stage. The method of measuring PH was different from the measure of PH in the mature period, which is the length of the main stem, measured from ground level to the tip of spike, excluding awns. However, the PH of seedling in this study was measured from ground level to the tip of the first leaf. We infer that the lower H^2^ for PH in this study was caused by the alterable length of the first leaf, as H^2^ for the length of wheat leaf was much lower that the PH in the mature period. Actually, it is hard to investigated the plant height before heading time.

Plant height is a complex trait, and is controlled by multiple major genes and micromajor genes. The QTLs detected in our research were micromajor genes, their heritability was lower, compared to the major genes.

We performed an ANOVA again, as was shown in table 1 below. The ANOVA results showed that variation of plant height was determined by genotype, environment and genotype × environment interactions (Table 2 in manuscript). Anyway, the lower H^2^ for PH in this study could be considered to be caused by the multi-environments.

**Table 1 - ANOVA analysis of RIL population in five different field environments.**

|  | Trait | Sum of squares | df | Mean square | F | Sig. |
| --- | --- | --- | --- | --- | --- | --- |
| Environment | PH(cm) | 156917.231 | 4 | 39229.308 | 1368.743 | .000 |
|  | TN(No./plant) | 15260.574 | 4 | 3815.143 | 780.237 | .000 |
|  | SFW(g/plant) | 249927.824 | 4 | 62481.956 | 144.401 | .000 |
|  | SDW(g/plant) | 4629.493 | 4 | 1157.373 | 161.466 | .000 |
| Genotype | PH(cm) | 81328.358 | 202 | 402.616 | 14.048 | .000 |
|  | TN(No./plant) | 14145.042 | 202 | 70.025 | 14.321 | .000 |
|  | SFW(g/plant) | 1227777.367 | 202 | 6078.106 | 14.047 | .000 |
|  | SDW(g/plant) | 121740.517 | 202 | 602.676 | 84.080 | .000 |
| Environment×genotype | PH(cm) | 46743.096 | 808 | 57.850 | 2.018 | .000 |
|  | TN(No./plant) | 8113.498 | 808 | 10.041 | 2.054 | .000 |
|  | SFW(g/plant) | 756089.686 | 808 | 935.755 | 2.163 | .000 |
|  | SDW(g/plant) | 38235.366 | 808 | 47.321 | 6.602 | .000 |
| Error | PH(cm) | 52363.345 | 1827 | 28.661 |  |  |
|  | TN(No./plant) | 8933.524 | 1827 | 4.890 |  |  |
|  | SFW(g/plant) | 790535.902 | 1827 | 432.696 |  |  |
|  | SDW(g/plant) | 13095.739 | 1827 | 7.168 |  |  |
| Total | PH(cm) | 8540199.707 | 2842 |  |  |  |
|  | TN(No./plant) | 460303.452 | 2842 |  |  |  |
|  | SFW(g/plant) | 13613684.720 | 2842 |  |  |  |
|  | SDW(g/plant) | 357908.221 | 2842 |  |  |  |

P13-L221-228.: The genetic map constructed in present study containing 54 segregation distortion loci. I suggested that the authors confirm whether these segregation distortion loci have an effect on QTL mapping.

Response:

In this study, 54 segregation distortion loci were detected, but these segregation distortion loci don’t affect QTL mapping. If the high density map was constructed accurately, and then the impact of segregation distortion on QTL analysis can be ignored. QTL IciMapping software can be used to construct genetic map, QTL mapping in segregation distortion population. And we checked the segregation ratio of the loci framed the QTLs and no significant segregation distortion was found. And the relevant results were described in the discussion of the manuscript (P21-L343-346 ans P22-L361-364).

P19-L290-293: The authors claim that the growing periods of two parents and RILs were consistent, but I found this claim was not supported by the presented data.
Response:

Sorry, the expression of this sentence is not accurate enough. Actually, the difference of growing periods between two parents and RILs exist, but was very small. And we revised the sentence, changed ‘consistent’ into ‘slight different’.
Reviewer #2: This manuscript reports important chromosomal positions of QTLs for four biomass traits that affect wheat yield and tolerance to abiotic stresses. It would be worthy of being published if the numerous typographical and grammatical errors (identified in the uploaded file) are corrected throughout the manuscript. It is also suggested that authors modify the figure of chromosomes 1D and 7D to depict the centromeres.

Response:

Thank you for your careful revision, suggestion on our manuscript, and recognition of our work. We have accepted your correction. We have tried our best to modify the manuscript. At the same time, this manuscript was edited for proper English language, grammar, punctuation, spelling, and overall style by one or more of the highly qualified native English speaking editors at NativeEE.

We have modified the figure of 1D and 7D, and attached the position of the centromeres (Fig 3).

6. PLOS authors have the option to publish the peer review history of their article ([what does this mean?](https://journals.plos.org/plosone/s/editorial-and-peer-review-process#loc-peer-review-history)). If published, this will include your full peer review and any attached files.
If you choose “no”, your identity will remain anonymous but your review may still be made public.
**Do you want your identity to be public for this peer review?** For information about this choice, including consent withdrawal, please see our [Privacy Policy](https://www.plos.org/privacy-policy).
Reviewer #1: No
Reviewer #2: Yes: Richard R.-C. Wang

[NOTE: If reviewer comments were submitted as an attachment file, they will be attached to this email and accessible via the submission site. Please log into your account, locate the manuscript record, and check for the action link "View Attachments". If this link does not appear, there are no attachment files to be viewed.]


While revising your submission, please upload your figure files to the Preflight Analysis and Conversion Engine (PACE) digital diagnostic tool, <https://pacev2.apexcovantage.com/>. PACE helps ensure that figures meet PLOS requirements. To use PACE, you must first register as a user. Registration is free. Then, login and navigate to the UPLOAD tab, where you will find detailed instructions on how to use the tool. If you encounter any issues or have any questions when using PACE, please email us at [figures@plos.org](mailto:figures@plos.org). Please note that Supporting Information files do not need this step.

Response:

Yumin Yang register a user(362072749@qq.com), and upload our the three figure files, they are valid TIF files.

Response to comments in manuscript by reviewer #1：

1.P9-L169-L171：”Independent of the differences between the two parents, in all trials there were significant variations in the investigated traits of the RIL populations, with values spanning much larger ranges than those defined by the parental values.” This sentence is little confusing.

Response:

We want to state the transgressive inheritance. so we added it to avoid confusion (P9-L171-L174).

2. P13-L212:”and then aligned by nnTwoOpt method.” ref should be added.

Response:

This sentence’s ref were added (P13-L214).

3.P20-L310：”Few QTLs for biomass have been identified in the diploid populations directly from *Ae. tauschii*, but in a hexaploidy genetic background.”

results or refs supporting this should be added.

Response:

We want to state that ter Steege et al haven’t detected QTLs for biomass in diploid populations of *Ae. tauschii*, but we detected in synthetic hexaploid wheat. This sentence added ref, and the sentence changed into “Few QTLs for biomass have been identified in the diploid populations of *Ae. tauschii* [37], but in a hexaploidy genetic background.”(P20-L313-L315).

Response to comments in manuscript by reviewer #2：

1. P7-L133-L135:The RILs and their parents Syn79 and Syn80 were executed on the Affymetrix platform of Axiom Wheat Breeder’s Genotyping Array with 13947 SNP markers including 1272 functional markers by China Golden Marker Biotech Co Ltd (Beijing, China).

“executed” need a better word.

Response:

We changed “executed” into “genotyped” (P7-L134)

2. P23-L380-L382: On the other hand, each of the synthetic wheat Syn79 and Syn80 having newly genes from tetraploid wheat and *Ae. tauschii* is a potential resource to broaden the genetic diversity for wheat breeding programs.

Newly acquired? Any evidence for the newly acquired genes in SHWs resulted from gene recombination following hybridization of tetraploid wheat and Ae. tauschii?

Response:

Sorry, the expression of “newly genes’ is not accurate enough, because we have no relevant evidence to confirm whether these genes have been reported. We can make sure that Syn80 has QTLs for enhancing early biomass in this study, with pleiotropic effects on plant height, tiller number, shoot fresh weight and shoot dry weight. These genes were elite genes from tetraploid wheat and *Ae. tauschii*. So we revised this sentence, and changed ‘newly genes’ into ‘ combining elite genes’ (P23-L386).
