# Supplementary material for: Mapping QTLs for enhancing early biomass derived from Aegilops tauschii in synthetic hexaploid wheat
Source: PLoS One. 2020 Jun 25;15(6):e0234882. doi: 10.1371/journal.pone.0234882 (PMC7316292; doi:10.1371/journal.pone.0234882)
Supplement: Attachment — Submitted filename: Response to Reviewers.doc [file pone.0234882.s007.doc]

Response to Reviewers

Dear Dr Zhang and Reviewers,

Thank you very much for giving us an opportunity to revise our manuscript, we appreciate editor very much for your careful guidance and timely communication, we will resubmit the manuscript according to your requirements.

Thanks the three reviewers for your recognition, positive and constructive comments and suggestions on our manuscript. We have made revision which marked in red in the paper.

We would like to express our great appreciation to you and reviewers for comments on our paper.

Looking forward to hearing from you.

Thank you and best regards.

Yours sincerely,

Wuyun Yang

Point-to-point reply to Reviewer #3:

1. The growing periods of RILs were the controlling factors for precise evaluation of the biomass related phenotypes, because the jointing stage was a relatively broad description. Please clarify it in M & M, for example, how many days after sowing to start phenotype evaluation, and how many days needed for finishing the plant height and tiller number evaluation and sampling for all the RILs?

Response: Actually, the mature period of each RIL was mostly similar (often much later than the local cultivars), and the difference between them was slight (Fig. S1-directly photographed from the field at May-11 2020). So, the phenotype evaluation could be collected at one time, as the jointing stage of them were also similar. In this study, Phenotypic data was investigated about 110 days after sowing, when the first internode came out, the plant height and tiller number evaluation were finished during sampling within 1-2 day. Thank you for your careful revision, this should be stated clearly in M & M.

Fig. S1 RILs planted in filed on 2020.

2. About the loci with segregation distortion. Please add the Chi-squared test of the most significant SNPs for each QTL in Table 6.

Response: We performed the Chi-squared test, as was shown in Table 6. For AX-94812958, the Chi-Square value is 38.506 (P<0.001). Chi-Square of AX-109908110 is 57.346 (P<0.001). the Chi-Square value of AX-110910133 is 6.821 (P<0.01). the Chi-Square value of AX-110605376 is 79.258 (P<0.001). the Chi-Square value of AX-109937582 is 82.713 (P<0.001). the Chi-Square of AX-109917900 is 76.722 (P<0.001).

Table S1 Observed number of parental genotypes at QTL loci.

|  | AX-94812958 | AX-109908110 | AX-110910133 | AX-110605376 | AX-109937582 | AX-109917900 |
| --- | --- | --- | --- | --- | --- | --- |
| Actual  range | 40 | 41 | 77 | 35 | 34 | 36 |
|  | 118 | 144 | 113 | 159 | 161 | 159 |
| Expected  range | 79 | 92.5 | 95 | 97 | 97.5 | 97.5 |
|  | 79 | 92.5 | 95 | 97 | 97.5 | 97.5 |

3 and 4. The language also need to be improved, for example, in line 43, change ‘the QTLs’ to ‘these two QTLs’; line 171, ‘There was transgressive inheritance.’; line 178, ‘these genes’ phenotypic traits’; line 193, ‘the gene related to early biomass has pleiotropic effects on all four traits.’; line 215-216, ‘There were no genetic gaps, with adjacent marker separation of no more than 50 cM occurring in each chromosome’; line 329, ‘indicating that QTLs for early biomass on 1DS in this population were new genes located in this study’. 4. Please modify the Figure legends and Table notes to make it easier for readers to understand. For example, line 264, ‘Positive and negative Syn79 and Syn80 alleles produced larger and smaller values, respectively.’

Response: Question 3 and 4 have been fixed in the text.

5. It will be good to add the phenotypic data and pictures of AT428 and AT333.

Response: Unfortunately, we did not introduce the AT428 and AT333 from CIMMYT. However, considering that synthetic hexaploid wheat Syn80 and Syn79 had the same tetraploid parent, their difference of early vigour originated from their diploid parent AT428 and AT333, and the early vigour of AT428 was supposed to be greater than AT333.
